# Integrated analysis of the redoxome and zinc proteome links ribosomal protein oxidation to zinc homeostasis

**DOI:** 10.1016/j.redox.2026.104152

**Published:** 2026-04-02

**Authors:** Katarzyna Jonak, Ulrike Topf

**Affiliations:** Laboratory of Molecular Basis of Aging and Rejuvenation, Institute of Biochemistry and Biophysics, Polish Academy of Sciences, Poland

**Keywords:** Aging, Zinc, Ribosomal proteins, Thiol oxidation, Oxidative stress

## Abstract

Zinc is an essential trace mineral for human health. However, consuming large amounts of zinc can be toxic. Therefore, zinc homeostasis must be actively regulated. Within cells, zinc mostly exists in a form bound with proteins. We show that the existence of zinc-binding proteins is a conserved feature of kingdoms of life, emphasizing the fundamental importance of zinc in biological systems. Cysteine residues chemically coordinate zinc binding within proteins and are highly sensitive to oxidative modifications under conditions of oxidative stress and aging. This study uses available datasets that analyzed the redoxome and combines this information with zinc-binding annotations. Our analysis reveals that zinc-binding cysteine residues are significant targets of reversible protein oxidation. In particular, we identified proteins of the cytosolic ribosome as zinc-binding oxidation targets during aging. We integrated our findings with data on changes in protein abundance under conditions of low zinc bioavailability. These analyses revealed that ribosomal proteins that bind zinc and are targets of oxidative modifications tend to be less abundant under zinc-depletion conditions. Additionally, the molecular dynamics simulations allowed us to link reversible oxidation of ribosomal proteins to zinc removal from these proteins and their following unfolding in zinc-deficient conditions. Thus, these findings open new possibilities for regulating zinc homeostasis during aging.

## 1 Introduction

Chronic oxidative stress is one of the hallmarks of aging, where the persistent generation of reactive oxygen species (ROS) or reactive nitrogen species (RNS) overwhelms the antioxidant defenses [1]. This imbalance accelerates aging by damaging DNA, lipids, and proteins. Less studied is the contribution of oxidative stress, particularly during aging, to changes in the balance of metal homeostasis. The interplay between zinc and redox homeostasis is bidirectional. Oxidative stress disrupts zinc-binding sites in proteins, releasing zinc ions that amplify oxidative damage. Low levels of zinc ions impair antioxidant enzymes, increase mitochondrial ROS production, and destabilize zinc-dependent proteins [2]. During aging, zinc levels decrease due to, among other factors, reduced dietary absorption in the elderly, which worsens immune function and increases susceptibility to infections [3].

Oxidation of cysteine residues is a pivotal chemical process in redox biology, modulating critical cellular processes, including proliferation, differentiation, and apoptosis [4,5]. The thiol (-SH) group of cysteine residues undergoes a spectrum of oxidative modifications that can be broadly categorized into reversible, such as disulfide bond formation, and irreversible, such as S-sulfinylation (-SO_2_H) and S-sulfonylation (-SO_3_H) [[6], [7], [8]]. Zinc ions form stable tetrahedral complexes with deprotonated cysteine thiolates (Cys-S^–^) [[9], [10], [11]]. Zinc coordination at cysteine sites reduces thiol nucleophilicity through Zn^2+^ Lewis acidity, polarizing the S–Zn bond and sterically shielding sulfur, thereby protecting against (over)oxidation by ROS or RNS in many proteins [[12], [13], [14]]. Thus, zinc may act as a conformational gatekeeper, balancing cysteine reactivity with redox signaling. Neighboring residues that surround redox-sensitive and zinc-binding motifs play a pivotal role in the protection of cysteine thiols. Acidic residues near zinc-binding sites, as seen in metallothioneins (MTs), stabilize zinc-thiolate clusters, while lysine-rich environments favor inducible zinc binding that competes with oxidation [15]. Reversible oxidation, meaning oxidation that can be switched back to the thiol state, coordinating cysteines to disulfides (-S-S-), sulfenic acids (-SOH), or S-nitrosothiols (-SNO), typically disrupts Zn–S bonds, favoring zinc dissociation in redox-responsive proteins. This may lead to polypeptide backbone reorganization, order-disorder transitions, alterations in the quaternary protein structure, and thus changes in protein function. Redox-active disulfides exist in alternate states and may act as dynamic redox switches within proteins [16].

The crosstalk between zinc-binding proteins (ZBPs) and redox-sensitive proteins is a significant aspect of molecular regulation in a cell. Particularly, it has gained attention through proteomics studies in different eukaryotic species [15,[17], [18], [19]] that mapped redox-sensitive proteins, identifying some of the oxidized cysteines as involved in zinc-binding motifs.

This work investigates the interplay between redox balance and zinc homeostasis, aiming to identify proteins that may mechanistically connect these two essential cellular processes during aging. To this end, we provide a systematic assessment of the evolutionary conservation of ZBPs and examine conserved chemical principles underlying zinc coordination. We then analyze the extent of overlap between zinc-binding and redox-sensitive proteins using publicly available datasets. Given that most organisms experience aging-associated increases in oxidative stress, it is plausible that redox modifications could influence zinc-binding capacity and, consequently, metal bioavailability. By integrating the information, we uncover a potential molecular node within the eukaryotic ribosome where redox regulation and zinc metabolism converge. Our findings suggest that reversible oxidation of cysteine residues in highly abundant ribosomal proteins could dynamically regulate zinc redistribution, potentially coupling redox status to translational control and cellular stress response during aging.

## 2 Results

### 2.1 Zinc-binding proteins across the tree of life

Zinc plays an essential structural, catalytic, and regulatory role in the functioning of the proteome across the tree of life [[20], [21], [22], [23]]. Our analysis of ZBPs and zinc-finger proteins (ZFPs) annotations from UniProt Knowledgebase (UniProtKB) identified approximately 12% of the human proteome as binding at least one zinc ion. These numbers align with previous studies, in which ∼10% of proteins were reported to bind zinc [15,21,24,25].

**Fig. 1:**
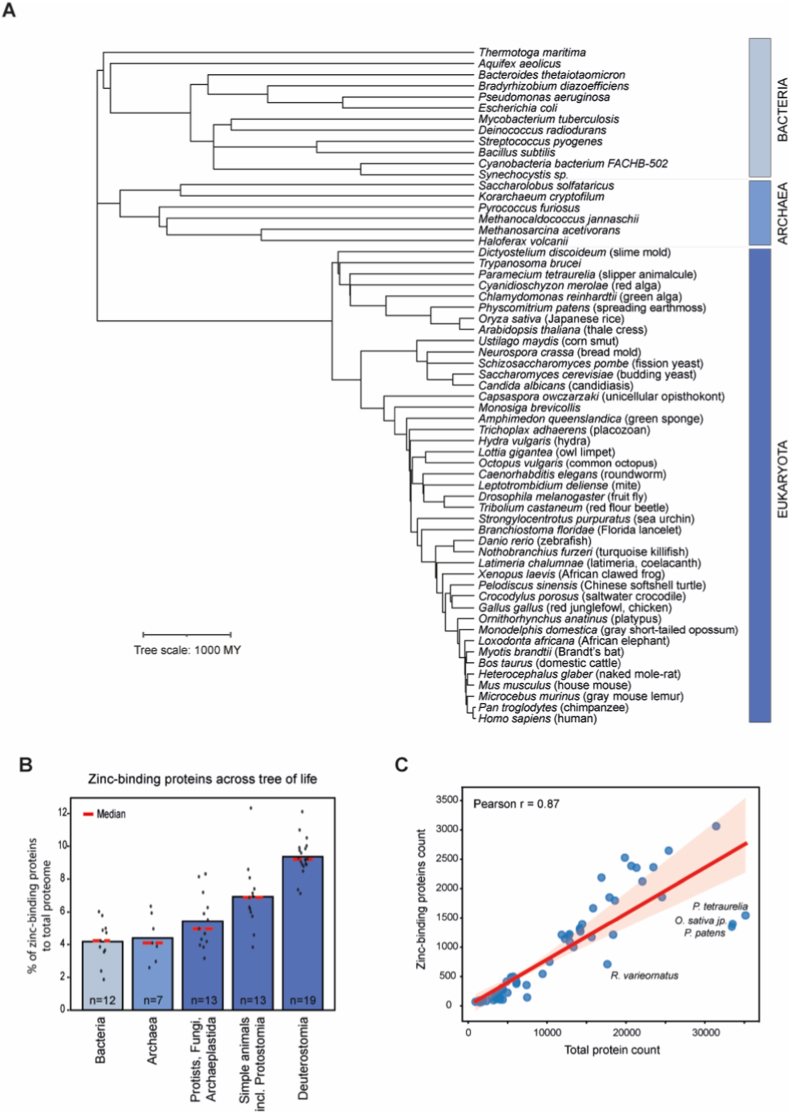
Comparative analysis of zinc-binding proteins across the tree of life. **(A)** Phylogenetic tree showing representative species from the three domains of life used in the meta-analysis: Archaea, Bacteria, and Eukaryota. Two eukaryotic species included in the study, *Ramazzottius varieornatus* (tardigrade) and *Schmidtea mediterranea* (planarian), and one archaeal, *Nitrosopumils martimus*, are not depicted (see “Methods”). The tree scale bar indicates 1000 million years (MY) of evolutionary time. **(B)** Average proportion of proteins annotated as “zinc-binding” in UniProtKB across proteomes of 64 species. The Eukaryota domain was divided into three groups: Protists, Archaeplastida and Fungi, simple animals including Protostomia, and Deuterostomia (includes vertebrates). Black dots – individual species values; red dashed lines – group medians; *n* – number of species in a group. **(C)** Correlation plot between the number of zinc-binding proteins and the number of proteins in each studied proteome. Blue dots – number of a single species; red line – the trend line fitted by linear regression; *r* – Pearson's correlation coefficient.

To address the evolutionary conservation of the ZBP landscape, we performed a meta-analysis of proteomes of 64 species across three domains of life. We accessed proteomes of chosen representative species using UniProtKB and filtered them to remove redundancy (see “Methods”). We focused on 12 species of Bacteria, seven of Archaea, and 45 of Eukaryota, among which three were grouped into Protists, five into Archaeplastida (algae + land plants), five into Fungi, eight into Protostomia, three simple Metazoa but not Protostomia, 19 into Deuterostomia, and two species bridging simple organisms with early animals: *Capsaspora owczarzaki* and *Monosiga brevicollis* (Fig. 1A). The quality check of the annotations identified, on average, ∼44% of proteins marked as “reviewed” in UniProtKB. Approximately 95% were manually curated for 14 species, among them archaea *Methanocaldococcus jannaschii* and *Korarchaeum cryptofilum*, bacteria *Escherichia coli* and *Bacillus subtilis*, representatives of fungi *Saccharomyces cerevisiae* and *Schizosaccharomyces pombe*, and eight eukaryotic species, including human *Homo sapiens* **(**Supplementary Figure 1A, Supplementary Table 1). Additionally, we identified over 60% of the annotated proteins, enabling the characterization of zinc-binding properties and their correlation with the total and zinc-binding proteome size (Supplementary Figure 1A).

We identified, on average, 6.65% of ZBPs across species. This percentage increases with the complexity of the organism (Fig. 1B, Supplementary Figure 1B, Supplementary Table 1). The average proportion of ZBPs in Deuterostomia species, including mostly vertebrates, is 9.5%, whereas in Protists and Algae it does not exceed 5%, with Fungi reaching up to almost 7%. Bacteria and Archaea, on average, have ∼4% of ZBPs. This is comparable with a previous study that identified zinc-binding in 8.8% of proteins in eukaryotic species and 6% in prokaryotes, with the difference potentially explained by a broader range of protein expression regulation in eukaryotes [24]. Interestingly, in our analysis, the fraction of identified ZBPs in humans and the octopus *Octopus vulgaris* is higher than in other species, reaching above 12%. It is surprising in the case of the octopus that contains a generally low percentage of annotated proteins in UniProtKB (Supplementary Figure 1A).

As previously reported, we observed that zinc-dependent metalloproteomes correlate with proteome size (Fig. 1C) [20,26]. A larger proteome is accompanied by a more complex regulatory machinery that controls protein expression and homeostasis [27]. Enrichment analysis of ZBPs reveals that they are primarily involved in regulating transcription, DNA repair, and epigenetic modifications (Supplementary Fig. 1C, Supplementary Tables 2 and 3). Proteins containing zinc-finger domains are the largest family of transcription factors in eukaryotes, crucial for gene expression and chromatin remodeling [23,[28], [29], [30]]. Among nine species with >60% proteins reviewed in UniProtKB (*M. jannaschii*, *B. subtilis*, *E. coli*, amoeba *Dictyostelium discoideum*, *S. cerevisiae*, *S. pombe*, *Arabidopsis thaliana*, *M. musculus*, *H. sapiens*), we identified human and mouse as species with >6% of proteins annotated with zinc-finger domains, which is more than 70% and 50% of total ZBPs in these respective species. In fungi, ZFPs account for ∼4% of the proteome, while the lowest percentage is observed in bacteria and archaea (<1%).

Processes related to proteostasis are among the highly enriched ones regulated by ZBPs in all three domains of life. The most prominent are proteins involved in the regulation of translation, ribosome biogenesis, and tRNA-related functions, as well as RNA processing, mRNA stability, protein modification, and processing involving ubiquitination and subsequent proteasome-dependent degradation pathways (Supplementary Figure 1C).

### 2.2 Evolutionarily conserved cysteine-rich proteins mediate zinc coordination

Analysis of ZincBind database [31], containing 3D structural data of ZBPs from the Protein Data Bank (PDB), shows that among 115,747 zinc-binding residues, approximately 40% are cysteines (Cys), 30% histidines (His), and 16% aspartate (Asp) or glutamate (Glu). Our analysis of zinc-binding sites provided by UniProtKB for 64 species identified similar fractions on average, with around 40% of Cys and His residues annotated for zinc binding in eukaryotes and a higher percentage of His residues binding zinc in bacteria (Supplementary Figure 2A).

The coordination geometry in zinc-binding sites is often tetrahedral, involving four amino acids that are spatially arranged to enclose the zinc ion. According to data collected by Ireland and Martin [31] from all PDB structures of resolution better than 3 Å, tetrahedral coordination in single-zinc binding sites is by far the most common (>67%), with coordination number of five or more being less common (∼23%) and coordination number of three being rare (<10%). Within the four-coordinate complexes, sites involving coordination by four cysteines are annotated as C4 type, which the ZincBind database finds to be most common, with sites containing three cysteines and one histidine (C3H1) or two cysteines and two histidines (C2H2) progressively rarer. We confirmed a high percentage of these three motifs in *S. cerevisiae* according to MetalPDB [32] (Supplementary Figure 2B). In *E. coli* and *H. sapiens,* it is less than 25% and ∼40%, respectively.

**Fig. 2:**
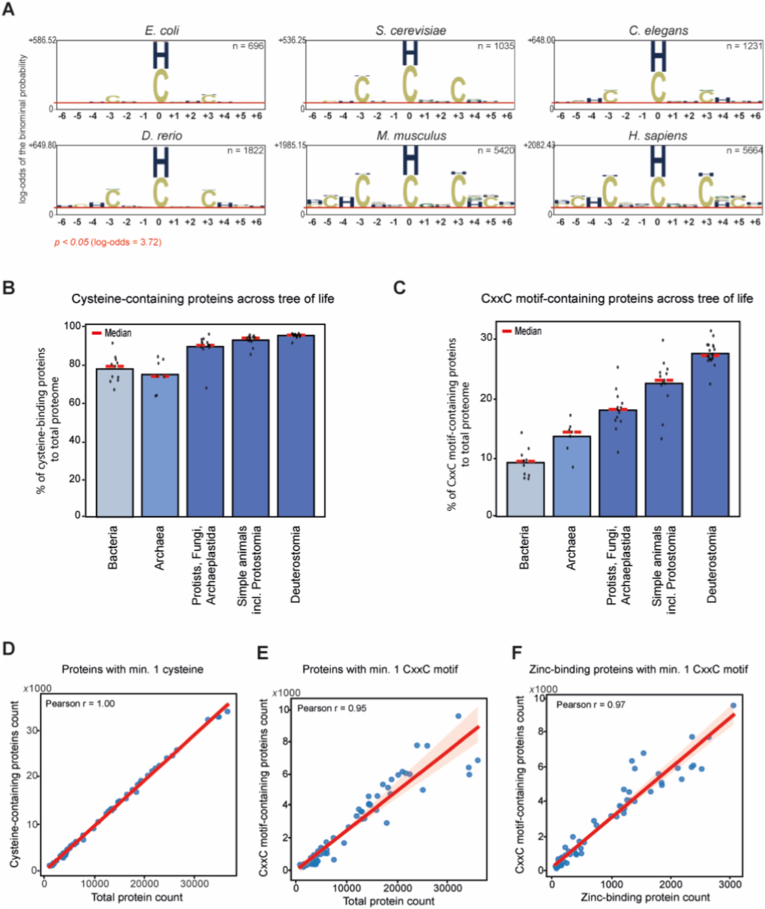
CxxC as a zinc-binding motif across species. **(A)** Enrichment analysis of amino acids involved in zinc binding based on UniProtKB annotations. Thirteen-amino-acid-long motifs, centered on zinc-binding residue (position “0”) from the UniProtKB “Binding site” dataset, were submitted for enrichment analysis against the entire proteome of each species. Six representative model species were selected, and the significantly overrepresented amino acids (p-value <0.05) are shown. *C* – cysteine, *H* – histidine; *n* – number of submitted peptides (motifs) as foreground. **(B–C)** Average proportion of zinc-binding proteins with cysteine residues (B) or at least one CxxC motif (C) across proteomes of 64 species. Black dots – individual species values; red dashed lines – group medians. **(D**–**F)** Correlation plots displaying relationships between: (D) the number of cysteine-containing proteins and total protein count per species, (E) the number of CxxC motif-containing proteins and total protein count, and (F) the number of CxxC motif-containing proteins and zinc-binding proteins. Blue dots – number of a single species; red lines – the trend line fitted by linear regression; *r* – Pearson's correlation coefficient.

Consistent with our analysis, enrichment analysis of 13-amino acid-long motifs with a central amino acid reported as zinc-binding in UniProtKB reveals cysteine and histidine as highly overrepresented residues involved in zinc ion coordination (Fig. 2A). We examined the cysteine content across the tree of life. More than 90% of the eukaryotic proteome contains at least one cysteine residue, compared to approximately 80% in bacteria and 70% in archaea (Fig. 2B). The number of proteins containing at least a single cysteine residue correlates strongly with the total proteome size (Fig. 2D).

The low number of proteins containing cysteine residues in prokaryotes correlates with the low number of proteins with more than a single cysteine present per protein (Supplementary Figure 3A, Supplementary Table 1). In both bacteria and archaea, more than 60% of proteins have a cysteine content of less than 1% of total protein sequence (0–**1% of Cys of amino acid sequence length), with the ancient bacteria Thermotoga martima**, UV radiation and oxidizing agents resistant bacteria **Deinococcus radiodurans**, as well as two archaea species, **Sulfolobus solfataricus** and **Pyrococcus furiosus,** with almost 75–**80% of proteins having Cys content below 1%. In comparison, average Cys content in a typical protein of eukaryotic species ranges from ∼1.1 per protein in Candida albicans** to >2.5 for **O. vulgaris**, **Danio rerio**, **M. musculus, Pan troglodytes** and **H. sapiens**. The more complex the organism, the more cysteines per protein are present (Supplementary Fig. 3A).

Cysteine residues are the backbone for metal-binding sites within classical C4 and C2H2 zinc-binding domains. These domains are often formed from two or one CxxC amino acid motifs, respectively, which means two cysteine residues (“C”) are separated by two other amino acids (“x”). Often located near alpha-helices, CxxC motifs may promote stable intra- or intermolecular disulfide bonds, enhancing protein stability in oxidizing environments [16]. CxxxC motifs also commonly bind zinc, as seen in ribosomal proteins like Rps26 (CxxxC-CxxC), providing flexible spacing for tetrahedral geometry while maintaining redox sensitivity.

The CxxC motif often acts as a redox-active motif in thioredoxin-fold enzymes (thioredoxins, glutaredoxins), where cysteines cycle through disulfide states independent of metal coordination to mediate thiol-disulfide exchange or peroxide detoxification [33,34]. The CxxC motif is present in Zn^2+^ binding sites, but also copper Cu^+^ chaperones, where they can form linear coordination with high affinity [[35], [36], [37], [38], [39], [40]]. While thermodynamically Zn^2+^ is preferential in tetrahedral geometry under physiological conditions, Cu^+^ binding dominates in copper-trafficking proteins due to softer ligands and reducing environments. In this work, we focus on the zinc coordination by the CxxC motif.

The CxxC motif is seen across different species, from *E. coli* to humans (Fig. 2A). There is a clear evolutionary trend: as we move from bacteria to fungi, plants, invertebrates, and vertebrates, both the cysteine content (Fig. 2B) and the number of CxxC motifs per protein increase (Fig. 2C). The CxxC motif is mostly prominent in Deuterostomia, including vertebrates (approximately 30% of proteins) (Supplementary Figure 3B). Among the CxxC motif-containing proteins, ∼20% contain 1 or 2 motifs, contrary to bacterial 10%. The highest fraction of such proteins (>5%) is observed in *O. vulgaris* and *D. rerio*. In comparison, unicellular eukaryote *S. cerevisiae* has 0.07% of such proteins, thale cress *A. thaliana* 0.4%, fruit fly *D. melanogaster* 1.36% and human ∼3%. This reflects increasing complexity in protein structure, redox regulation, and possibly signaling functions that involve cysteine residues and disulfide bonds. Similarly, to the number of cysteine sites, there is a strong correlation between the number of CxxC motifs and total protein count per species (Fig. 2E). The strong correlation between the number of proteins with at least a single CxxC motif and the annotation for binding of at least a single zinc ion (Fig. 2F) supports the motif analysis (Fig. 2A) and the notion of frequent involvement of this motif in zinc coordination. The majority of eukaryotic proteins bind zinc through the CxxC motif, although it is rarely the case for bacteria and archaea (Supplementary Figure 3C).

### 2.3 Zinc-binding proteins are preferentially oxidized at the early stage of aging

Recently, we characterized the reversibly oxidized cysteine-containing peptides of proteins during early stages of chronological aging in budding yeast *S. cerevisiae*, using the OxICAT method [41] for labeling of modified thiols along with mass spectrometry (MS) for quantification of levels of oxidation of detected peptides [19]. Here we refer to reversible oxidation as oxidized cysteine sites that, upon TCEP (tris(2-carboxyethyl)phosphine) treatment, can be reduced back to thiols. Our approach offered important insights into the molecular underpinnings of aging and the kinetics of proteome oxidation during aging, suggesting a hierarchy pattern of proteome oxidation under stress conditions. The study noted that oxidation-sensitive proteins were enriched for zinc ion binding.

**Fig. 3:**
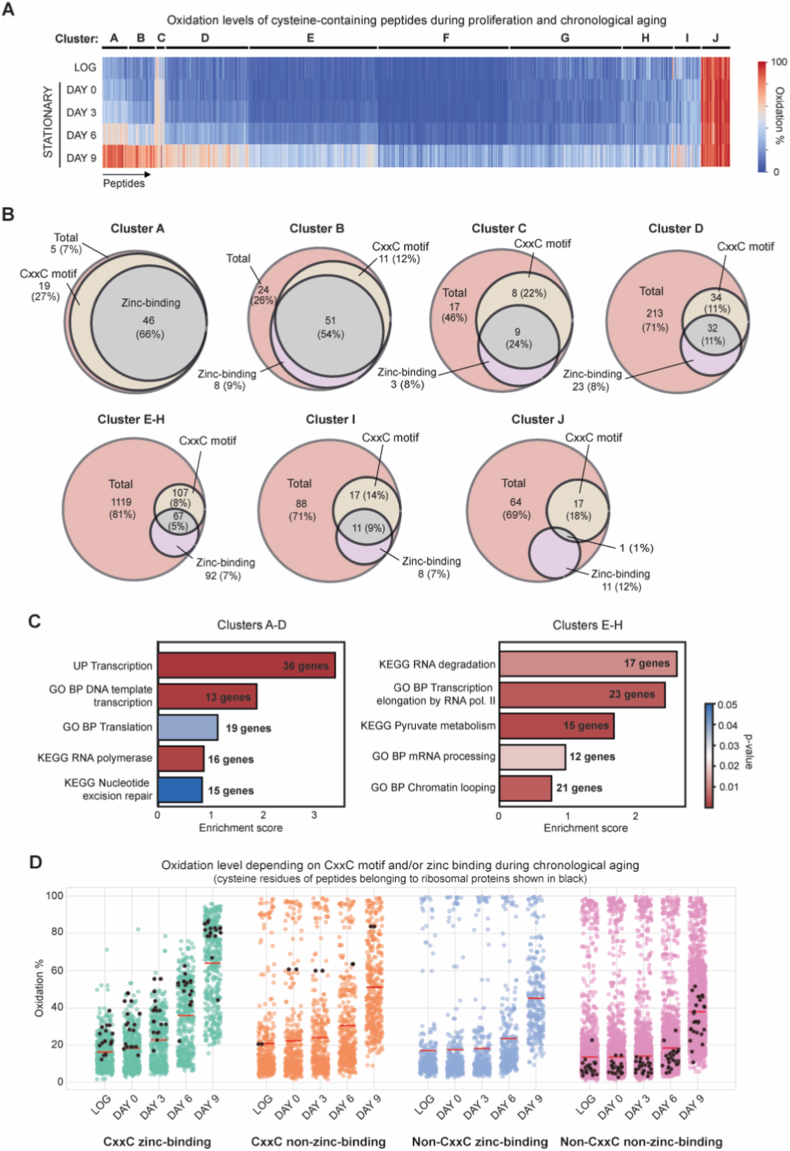
Redox modifications of zinc-binding proteins during yeast chronological aging. Data on reversible protein oxidation from chronologically aged yeast *S. cerevisiae* were obtained from Jonak et al. [19]. Yeast cultures were grown on minimal medium with 2% glucose. Samples were collected at approximately 6 h post-inoculation, representing the logarithmic growth phase (LOG), with actively proliferating cells, and at subsequent time points marking the onset and progression of chronological aging: day 0 (42 h after LOG sampling) and days 3, 6, and 9. Day 0 is characterized by diauxic shift (adaptation from fermentation to respiration). Days 0 and 3 are classified as “early aging”, day 6 as “middle aging”, and day 9 as “late aging”. (A) Heatmap illustrating the level (percentage, %) of reversible oxidation of cysteine-containing peptides across time points of chronological aging. Peptides are grouped into eight clusters based on their oxidation profiles: A-C – early oxidation, D – middle-point oxidation, E-H – late oxidation, I – mixed responses, J – highly oxidized all the time. **(B)** The number of zinc-binding proteins and CxxC motif-containing proteins identified as reversibly oxidized during different stages of yeast chronological aging. **(C)** Functional enrichment analysis of zinc-binding proteins from early- and middle-oxidation clusters A-D and late-oxidation clusters E-H. Bar plots display representative annotation terms (selected for the highest gene counts and lowest *p*-values) of the five annotation clusters with the highest enrichment score. The number of genes per annotation cluster is indicated, with colors representing significance at *p* < 0.05. The background gene set for enrichment was defined as all genes included in the corresponding oxidation clusters. UP – UniProtKB, GO BP – Gene Ontology Biological Process. **(D)** Levels of oxidation (%) of cysteine residues belonging to quantified peptides at different stages of chronological aging according to CxxC motif presence and zinc-binding status. Cysteine residues of cytoplasmic ribosomal proteins are shown as black dots. Red line, median of peptides' oxidation per group.

Based on the kinetics of the oxidation of particular cysteine-containing peptides, we clustered the peptides into ten groups (Fig. 3A) [19]. Importantly, a single cysteine-containing peptide can be assigned only to a single cluster, while a single protein with more than one peptide quantified can be assigned to multiple clusters based on the assignment of these peptides. We refer to proteins with at least one cysteine-containing peptide grouped within clusters A-C as “early oxidized proteins”, and proteins within cluster D as “middle-aged oxidized”. These early and middle-aged oxidized proteins exhibit a normal abundance distribution, indicating their increased oxidation signals arise independently of higher protein abundance (Supplementary Figure 4A). Clusters E-H contain peptides with a low oxidation state throughout aging, with an increase observed at the late stage of aging (day 9). Cluster J contains peptides characterized by a constantly high oxidation state of above 70%, such as peptides of mitochondrial proteins. Cluster I contains a mix of behaviors. Our data demonstrate that proteins oxidized early in chronological aging predominantly contain CxxC motifs, which is a canonical zinc-binding motif. This is in agreement with previous data on proteome oxidation throughout aging in various species [17,[42], [43], [44]] (Supplementary Figure 4C). Due to the greater amount of available data on zinc binding and response to zinc deficiency, similar techniques used to characterize oxidation levels (OxICAT), and the possibility to study the kinetics of changes in proteome oxidation during aging, we focused our further analysis on budding yeast datasets related to oxidation during chronological aging and stress conditions.

To obtain more detailed information on zinc-binding proteins, we combined four datasets containing annotations from UniProtKB (along with GO Molecular Function terms), from MetalPDB [32], Andreini et al. [45], and Wang et al. [46] (Supplementary Fig. 4D, Supplementary Table 4). Notably, out of 184 early oxidized proteins, 103 are zinc-binding, with the highest percentage in cluster A above 65% (Fig. 3B). All of the zinc-binding proteins within these clusters contain the CxxC motif. This contrasts with the middle (cluster D) and late aging stages (clusters E-H), where fewer oxidized proteins bind zinc: in middle age, it is 32 out of 302, and this proportion decreases further as aging progresses. This pattern suggests a strong association of zinc-binding with early oxidation events.

The early- and middle-age oxidized proteins (clusters A-D) are largely non-enzymatic and mostly critical for regulating proteostasis [19]. Our analysis of 458 zinc-binding proteins from these clusters shows the highest enrichment in processes regulating transcription, DNA repair, and translation (Fig. 3C, left). Clusters containing 159 ZBPs oxidized late during aging are enriched for processes related to RNA stability and processing (Fig. 3C, right).

Next, we asked whether there is a specific trend of reversible oxidation among cysteine residues of proteins based on zinc-binding capacity and the presence of CxxC motif/s during chronological aging. We observed a general trend of ZBPs containing CxxC motif/s of average basal oxidation below 20% with a steady increase to almost 40% at day 6 and above 60% at day 9 (Fig. 3D). Ribosomal proteins were among the proteins with higher oxidation levels during each state of aging (day 0: ∼30%, day 3: ∼36%, day 6: ∼49%, day 9: ∼79%), which is consistent with the enrichment analysis of early-oxidized clusters and our previously reported analysis [19]. The ribosomal proteins represent the highly abundant fraction of the proteome, yet their abundance does not influence oxidation levels at early aging stages, as late-oxidized ribosomal proteins are within the highly abundant proteins quantified by MS (Supplementary Figure 4B). The elevated oxidation reflects redox sensitivity rather than a detection bias from higher abundance. Interestingly, within the early-oxidized group, we see two cysteine sites belonging to a single peptide of a ribosomal large-subunit protein, Rpl34, that were not annotated as zinc-binding. However, the presence of two CxxC motifs indicates the possibility of zinc-binding. We did not find any ribosomal proteins that were annotated as binding zinc independently of a CxxC motif. Ribosomal proteins that do not bind zinc and lack the CxxC motif in their sequences exhibited low levels of oxidation on their cysteine sites, except during late aging (day 9), where they clustered below the average proteome oxidation level.

### 2.4 Oxidatively stressed and aged cells have overlapping redox regulation targets

To understand if the early oxidized proteins during aging respond similarly to other conditions leading to oxidative stress, we compared redox dynamics of proteins under both chronological aging [19] and acute oxidative stress [18]. The second dataset consists of two subsets on proteome oxidation during logarithmically growing yeast cells under exogenous stress caused by hydrogen peroxide (H_2_O_2_) treatment and under endogenous stress caused by mutation in the mitochondrial protein import factor Mia40 (Supplementary Fig. 5A and B).

**Fig. 4:**
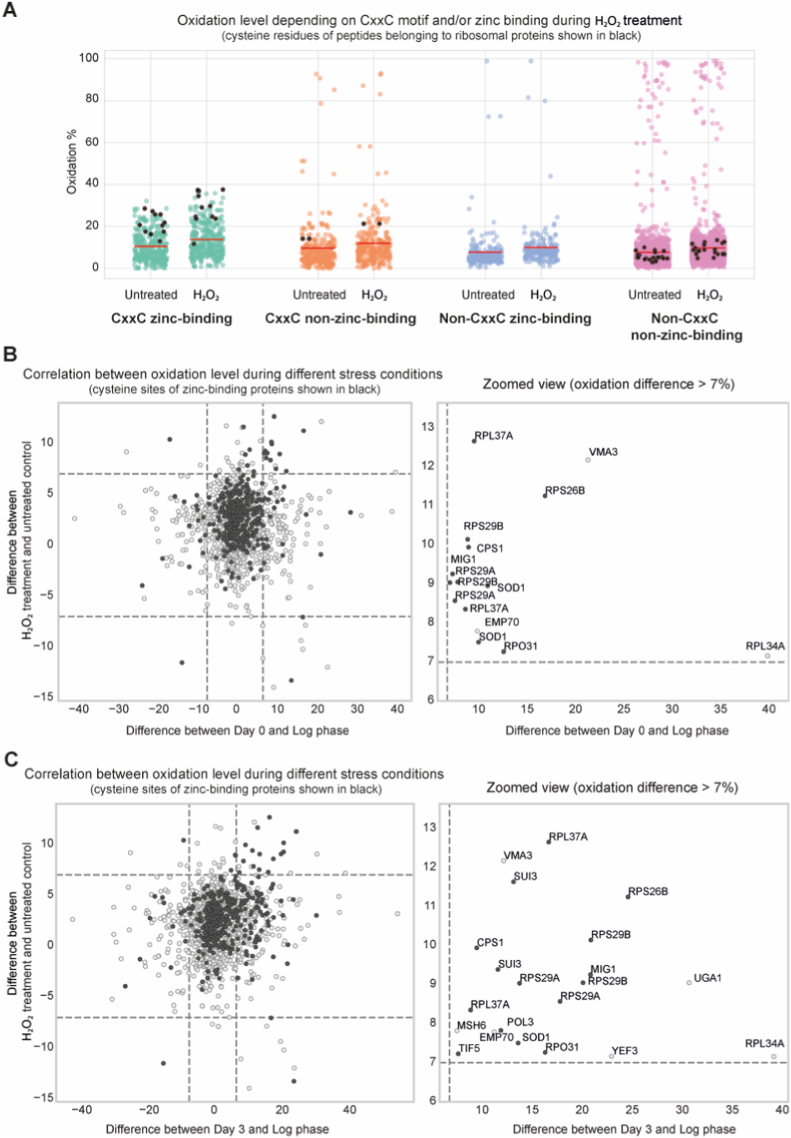
Redox modifications of zinc-binding proteins during yeast chronological aging and H_2_O_2_-induced oxidative stress. Data on chronologically aged yeast was obtained from Jonak et al. [19], while data on H_2_O_2_-induced stress of logarithmically grown yeast from Topf et al. [18] **(A)** Oxidation levels (%) of cysteine residues of quantified peptides in yeast upon H_2_O_2_-treatment and in untreated control cells. Data are categorized according to the presence of the CxxC motif and zinc-binding annotation. Cysteine residues of cytoplasmic ribosomal proteins are shown as black dots. Red line, median of peptides' oxidation per group. **(B–C)** Correlation analyses comparing oxidation changes of individual cysteine residues of quantified peptides between early stages of aging and H_2_O_2_ treatment. **(B)** Changes relative to day 0 of aging (diauxic shift). **(C)** Changes relative to day 3 of aging (early aging phase). Cysteine residues belonging to zinc-binding proteins are highlighted in black.

Firstly, we focused on comparing the aging dataset with data on H_2_O_2_-treated cells (Fig. 4). The initial aging dataset consists of 3564 cysteine sites of 1772 proteins in total. Among them, 2663 cysteine sites of 1535 proteins are quantified also in the H_2_O_2_-treatment dataset, among which 238 proteins are annotated as zinc-binding (Supplementary Figure 5A). We observed that, similarly to the aging dataset, there is the strongest response to oxidative stress in proteins annotated as zinc-binding with the CxxC motif present, with ribosomal proteins being amongst the most highly oxidized, above the average of that group (Fig. 4A). We next correlated the oxidation levels of cysteine sites common to the dataset on early aging (day 0 and day 3) and H_2_O_2_ treatment (Fig. 4B and C). This comparison helped reveal conserved and differential redox responses in proteins related to aging and stress. Among the proteins with oxidation increase above 7% compared to the control cells (untreated for H_2_O_2_ treatment and LOG for aging dataset), we identified 11 proteins in H_2_O_2_-treatment vs. day 0 (Fig. 4B) and 17 in H_2_O_2_-treatment vs. day 3 (Fig. 4C). In both early-aging days, we identified 5 common ribosomal proteins, all containing CxxC motifs and, apart from Rpl34, annotated as zinc-binding. If we consider clustering into A-J groups of oxidation trends during aging [19], we identify 16 early-aged oxidized proteins with oxidation increase >7% upon H_2_O_2_-treatment. Among them, 13 are annotated as zinc binding.

**Fig. 5:**
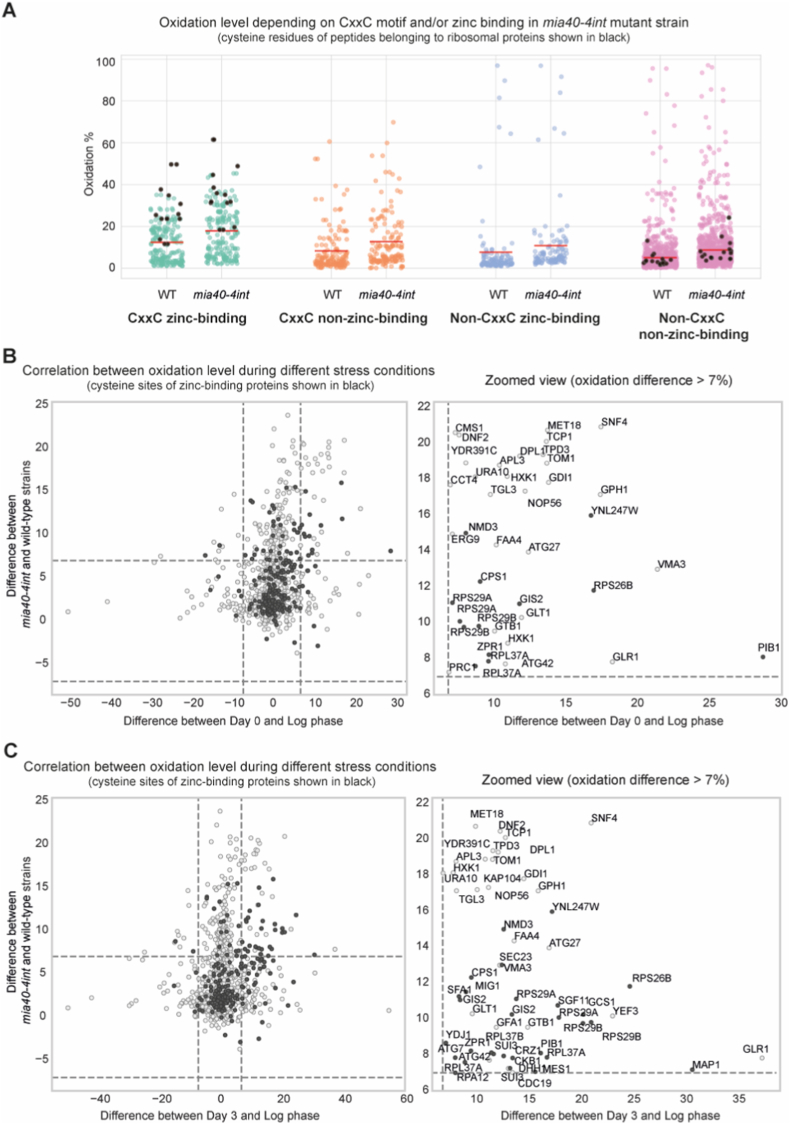
Redox modifications of zinc-binding proteins during yeast chronological aging and in endogenously stressed *mia40-4int* mutant yeast. Data on chronologically aged yeast was obtained from Jonak et al. [19], while data on *mia40-4int* mutant cells grown at logarithmic phase from Topf et al. [18]. **(A)** Oxidation levels (%) of cysteine residues of quantified peptides in wild type (WT) and *mia40-4int* mutant yeast cells. Data are categorized according to the presence of the CxxC motif and zinc-binding annotation. Cysteine residues of cytoplasmic ribosomal proteins are shown as black dots. Red line, median of peptides' oxidation per group. **(B–C)** Correlation analyses comparing oxidation changes of individual cysteine residues of quantified peptides between early stages of aging and in *mia40-4int* mutant cells grown at logarithmic phase. **(B)** Changes relative to day 0 of aging (diauxic shift). **(C)** Changes relative to day 3 of aging (early aging phase). Cysteine residues belonging to zinc-binding proteins are highlighted in black.

Next, we analyzed the correlation between the response to stress during aging and the response resulting from mitochondrial dysfunction, elevating endogenous ROS. To analyze this, we used data on a thermo-sensitive mutant of Mia40 translocase, *mia40-4int*, that exhibits mitochondrial dysfunction and increased oxidative stress [[47], [48], [49], [50]]. We observed a similar pattern as for H_2_O_2_-treated cells, with a lower total number of proteins identified as redox sensitive in both datasets, but a higher number of proteins with >7% increase in oxidation in comparison to the appropriate control samples (Fig. 5). 1499 cysteine sites of 1061 proteins in total are quantified in the *mia40-4int* and aging datasets, among which 186 proteins are annotated as zinc-binding (Supplementary Figure 5B). Again, we observed the strongest response in reversible oxidation of proteins annotated as zinc-binding with CxxC motif/s (Fig. 5A). With the ribosomal proteins having a higher oxidation level than the average of the group, we conclude that they are highly sensitive to redox changes independently of the source of ROS. Correlation analysis revealed 4 and 5 ribosomal proteins oxidized >7% in *mia40-4int* mutant cells in comparison to the wild-type cells, and on days 0 and 3 of aging, respectively, compared to the LOG culture (Fig. 5B and C). In total, there are 36 proteins with oxidation increase above 7% in datasets of *mia40-4int* mutant cells and aging day 0 cells (Fig. 5B). If we compare *mia40-4int* to day 3, this number increases to 51. Looking specifically at early-oxidation clusters (A-C), we identify 44 common proteins between this group and proteins with an increase in peptides’ oxidation >7%, among which 21 are zinc-binding, and 5 are ribosomal proteins.

### 2.5 Zinc-binding in ribosomal proteins with the CxxC motif occurs across eukaryotes

Cytosolic ribosomal proteins containing CxxC motif(s) are evolutionarily conserved among eukaryotes (Table 1), which indicates their functional importance. Nine proteins, Rpl34/eL34, Rpl37/eL37, Rpl40/eL40, Rpl42/eL42, Rpl43/eL43, Rps26/eS26, Rps27/eS27, Rps29/uS14, and Rps31/eS31, maintain the motif in diverse eukaryotic species, though some variations exist in organisms like *C. elegans,* and there is no consensus about their zinc-binding capabilities. In some species, some ribosomal proteins exist as closely related paralogs.

**Table 1:** **Evolutionarily conserved cytosolic ribosomal proteins containing at least one CxxC motif.** Comparison was performed between six model organisms. Orthologs of CxxC motif-containing cytosolic ribosomal proteins were shown, indicating the annotated zinc-binding as “+Zn”, according to UniProtKB and PDB files. Proteins that do not contain CxxC motif/s but are orthologs of motif-containing proteins in other species are marked with a star “∗”. A unified nomenclature for ribosomal proteins across species was proposed by Ban et al., 2014 [51].

*S. cerevisiae*	*C. elegans*	*D. melanogaster*	*D. rerio*	*M. musculus*	*H. sapiens*	Unified nomenclature
RPL34 A/B	rpl-34∗	RpL34a, RpL34b	rpl34	Rpl34 (+Zn)	RPL34 (+Zn)	eL34
RPL37 A/B (+Zn)	rpl-37.1, rpl-37.2 (+Zn)	RpL37-1, RpL37-2 (+Zn)	rpl37 (+Zn)	Rpl37(+Zn)	RPL37 (+Zn)	eL37
RPL40 A/B (+Zn)	ubq-2	RpL40	uba52 (+Zn)	Uba52 (+Zn)	UBA52 (+Zn)	eL40
RPL42 A/B (+Zn)	rpl-36A	RpL36A	rpl36a	Rpl36a, Rpl36al (+Zn)	RPL36A (+Zn)	eL42
RPL43 A/B (+Zn)	rpl-37A/rpl-43 (+Zn)	RpL37A (+Zn)	zgc:171772 (rpl37a)	Rpl37a (+Zn)	RPL37A, RPL37AP8 (+Zn)	eL43
RPS26 A/B (+Zn)	rps-26	RpS26	rps26, rps26l	Rps26 (+Zn)	RPS26(+Zn)	eS26
RPS27 A/B (+Zn)	rps-27 (+Zn)	RpS27 (+Zn)	rps27.1, rps27.2, rps27l (+Zn)	Rps27, Rps27l, Rps27rt	RPS27, RPS27L (+Zn)	eS27
RPS29 A/B (+Zn)	rps-29 (+Zn)	RpS29 (+Zn)	rps29 (+Zn)	Rps29 (+Zn)	RPS29 (+Zn)	uS14
RPS31 (+Zn)	rps-27A/ubl-1 (+Zn)	RpS27A (+Zn)	rps27a	Rps27a	RPS27A, RPS27AP5 (+Zn)	eS31
RPS12∗	rps-12	RpS12∗	rps12∗	Rps12∗	RPS12∗	eS12

**Fig. 6:**
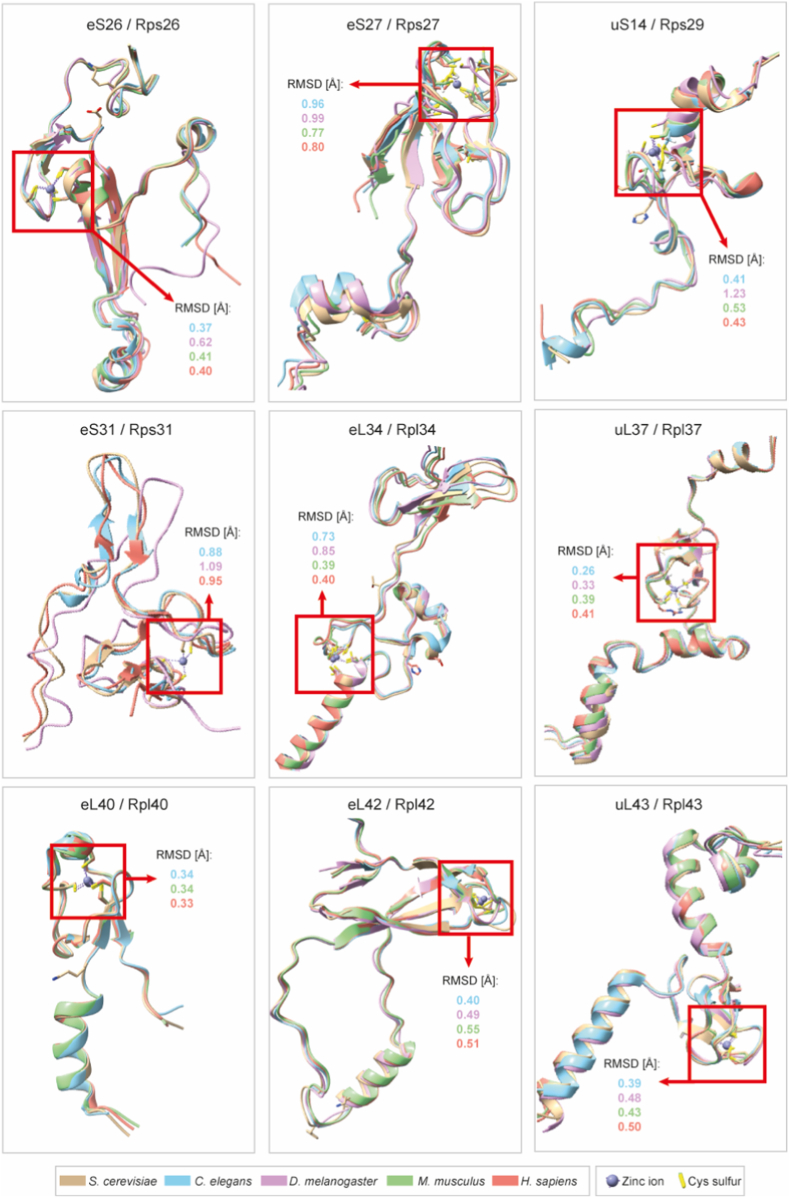
Comparison of structures of CxxC motif-containing ribosomal proteins across species. Ribosomal proteins from five species were used: *S. cerevisiae* (beige; PDB: 4U3M), *C. elegans* (blue; PDB: 9BH5), *D. melanogaster* (purple; PDB: 6XU8), *M. musculus* (green; PDB: 7CPU), and *H. sapiens* (red; 9PA7). For yeast, paralogs Rps26B, Rps27A, Rps29A, Rps31, Rpl34A, Rpl37A, Rpl40, Rpl42A, and Rpl43A are shown. Cysteine's sulfurs are shown as yellow rods. RMSD (root mean square deviation) of the zinc-binding pocket was calculated (for Cα atoms of±6 residues around each of four cysteines) between yeast ribosomal proteins and their orthologs in other species. RMSD is given in Angstrom (Å).

To analyze the possibility of zinc-binding by the CxxC-containing ribosomal proteins across eukaryotes, we aligned the solved structures of these proteins coming from the selected eukaryotic species (Fig. 6). The alignment reveals strong superimposition of the C4 zinc-binding domains with an RMSD (root mean square deviation) of the cysteine sites and surrounding folds <1 Å for most of the tested ribosomal proteins. Low RMSD implies conserved zinc binding affinity and functional geometry transferable across eukaryotes.

We inspected the aligned structures for zinc binding and found that each of these ribosomal proteins can bind a zinc ion, although it is not observed in all species. For example, Rpl34/eL34 shows no zinc binding in yeast, but newly solved mouse and human structures reveal clear zinc coordination. Complementary modeling with Metal3D [52] of yeast Rpl34A solved without zinc ion (Supplementary Figure 6A) predicts high-affinity zinc binding, suggesting a latent zinc-binding capacity that may be physiologically regulated rather than absent. Notably, *C. elegans* and *D. melanogaster* structures usually lack zinc ions. More structures have been solved and are accessible in PDB for *S. cerevisiae*, *M. musculus*, and *H. sapiens* than for the other two species, which may explain the increased prevalence of zinc coordination in the former. In some ribosomal proteins, zinc is absent in certain species, likely due to crystallographic challenges such as specific purification and crystallization procedures, low occupancy of the zinc ion under aerobic conditions, insufficient resolution to resolve the metal site, or omission of zinc ions due to other research focuses.

Phylogenetic tree analyses of CxxC motif-containing ribosomal proteins further demonstrate exceptional evolutionary conservation across eukaryotes, with short branch lengths (<0.25 substitutions/site) and tight ortholog clustering (Supplementary Fig. 6B).

An interesting observation emerges when comparing CxxC motif-containing ribosomal proteins between eukaryotes and bacteria. For example, Rpl37/eL34 contains two conserved CxxC motifs in eukaryotes but lacks a bacterial homolog, indicating that this zinc-binding feature likely evolved after the divergence of eukaryotes and bacteria. In contrast, eukaryotic Rps29/uS14 has a bacterial homolog that exists in two paralogous forms. One form, S14BsC^+^, binds zinc through a CxxC motif, whereas the other paralog expressed under zinc-limiting conditions, S14BsC^−^, lacks this motif and does not bind zinc [53,54].

### 2.6 Linking oxidative modifications of ribosomal proteins to zinc release

To investigate the link between cysteine oxidation and ribosomal protein rearrangement involving zinc release, we employed a multiscale computational modeling on four representative ribosomal proteins with three or four cysteine residues identified as early oxidation targets during budding yeast chronological aging [19]. These included large subunit proteins Rpl34 and Rpl37, and small subunit proteins Rps26 and Rps31 (a ubiquitin-ribosomal fusion protein).

**Fig. 7:**
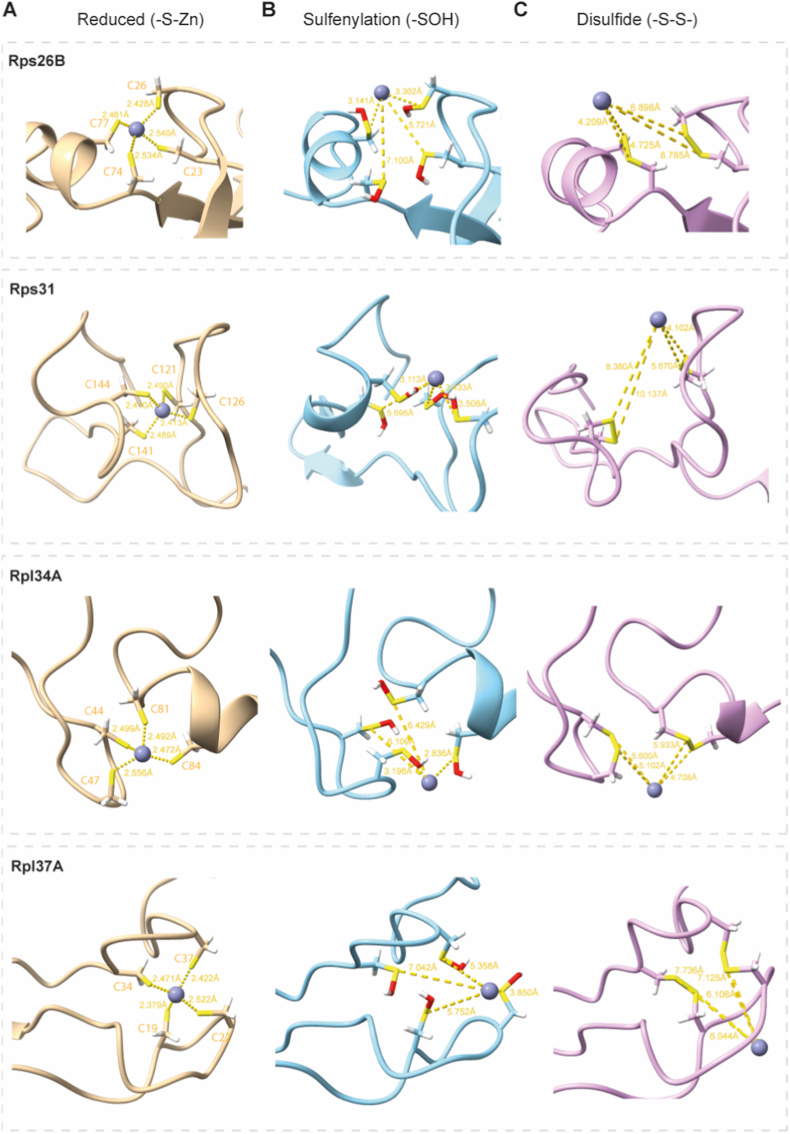
Zinc repulsion during oxidation of cysteine residues of chosen yeast ribosomal proteins. Final structures of the MD simulations run for 0.5 ns, depicting zinc release as a fast and early response to oxidation. **(A)** Models of zinc binding in structures with deprotonated cysteines (-S-Zn; beige). **(B)** Models of zinc repulsion in structures with sulfenylated cysteines (-SOH; blue). **(C)** Models of zinc repulsion in structures with disulfide bonds (-S-S-; pink). Distances between zinc ions and cysteine sulfurs are shown as yellow dashed lines, and the values are given in Angstroms (Å). Yellow rods are sulfur atoms, red are oxygen, and white are hydrogen atoms.

We applied quantum mechanical calculations and molecular dynamics (MD) simulations [[55], [56], [57], [58]] to compare zinc coordination in the reduced state versus reversible oxidative modifications: sulfenylation (-SOH) and disulfide bond formation (-S-S-; predicted disulfide positions [59]). In control simulations with reduced cysteines, zinc remained tightly bound in a tetrahedral C4 geometry for all four ribosomal proteins, with average distances between zinc ion and sulfur atoms between 2.3 Å and 2.6 Å (Fig. 7A, Supplementary Fig. 7A). Oxidative modifications, however, triggered a cascade of destabilization. Sulfenylation initially disrupted coordination by introducing steric hindrance from the –OH group and shifting the partial negative charge on sulfur, weakening the electrostatic interactions between zinc and sulfur (Fig. 7B, Supplementary Fig. 7B). The trajectory in MD simulations clearly shows the instability of the zinc binding, where it moves away from the sulfurs of reactive cysteines (Fig. 7C, Supplementary Fig. 7C). Disulfide formation further enforced conformational rigidity, covalently pairing cysteines and pulling their sulfur atoms >4 Å apart from zinc, beyond the binding threshold. Disulfides covalently lock cysteine pairs, physically withdrawing the sulfur atoms from the coordination geometry.

In all four tested proteins, these modifications resulted in a "mechanical ejection" of the zinc ion, possibly compromising site integrity and implying downstream effects on protein stability under zinc-limiting conditions. Thus, we further tested the protein's structural stability by performing MD simulations for 10 ns in the absence of zinc in the oxidative conditions for a representative protein, yeast Rps26B. The structure of the whole protein is unstable during the simulation, resulting in >15 Å RMSD difference between the energy-minimized structure at the beginning of the simulations and the final frame (Supplementary Figure 8A). The cysteine pocket is visibly opening, making the hydrophilic patch more accessible to the solvent (Supplementary Fig. 8B and C). The expansion of the pocket may be driven by a significant loss of metallic tethering, most notably reflected in the Cys26-Cys77 distance, which nearly doubled from 4.96 Å to 9.87 Å (Supplementary Figure 8D). Next, we calculated the solvent accessible surface area (SASA) of the cysteines that participate in the zinc binding. Removal of the coordinating zinc ion and subsequent cysteine oxidation triggered a significant global conformational expansion, evidenced by a 974.88 Å^2^ increase in total protein SASA. Within this transition, the cysteine cluster experienced a 38.7% increase in exposure (expansion of 49.49 Å^2^), while the total surface area of the isolated cluster expanded by ∼10% (66.44 Å^2^) (Supplementary Figure 8E). These metrics suggest that while the protein maintains significant shielding of the site, the oxidation of the sulfur atoms and subsequent loss of zinc ion forces a structural loosening that shifts the cluster toward a more disordered and solvent-accessible state. The resulting open state likely increases the accessibility of previously buried regions, which may facilitate further solvent interactions, downstream signaling events, and possible subsequent protein degradation.

### 2.7 Dynamics of ribosomal proteins in response to oxidative stress and zinc deficiency

Zinc-deficient growth conditions in yeast result in a significant increase in ROS [60,61], providing a simplified, controlled model for investigating the zinc-redox axis. This system allows us to examine changes in ZBPs levels under conditions that mimic key redox features of aging. In budding yeast, zinc deficiency (1 μM ZnCl_2_ in low-zinc medium) induces profound remodeling of the proteome, as shown by Wang et al. [46]. Our re-analysis of the data shows that during a time course in zinc-deficient medium in logarithmically-grown culture, approximately 1088 proteins increase in abundance 16 h after shifting to zinc-deficient medium, while only 343 decrease in abundance (Supplementary Fig. 9A and B, Supplementary Table 5). We refer to up- or downregulated proteins as proteins with a fold change relative to time point 0 of >0.5 or < -0.5, respectively. As time progresses in zinc-deficient medium, there is a shift toward the downregulation of ZBPs, following the global proteomic pattern. This shift aligns with the zinc-sparing strategy, characterized by the downregulation of zinc-intensive processes and the further upregulation of stress adaptation pathways [15,46]. Proteins that decrease in abundance (fold change < -0.5) predominantly include cytosolic ribosomal proteins (83 proteins).

**Fig. 8:**
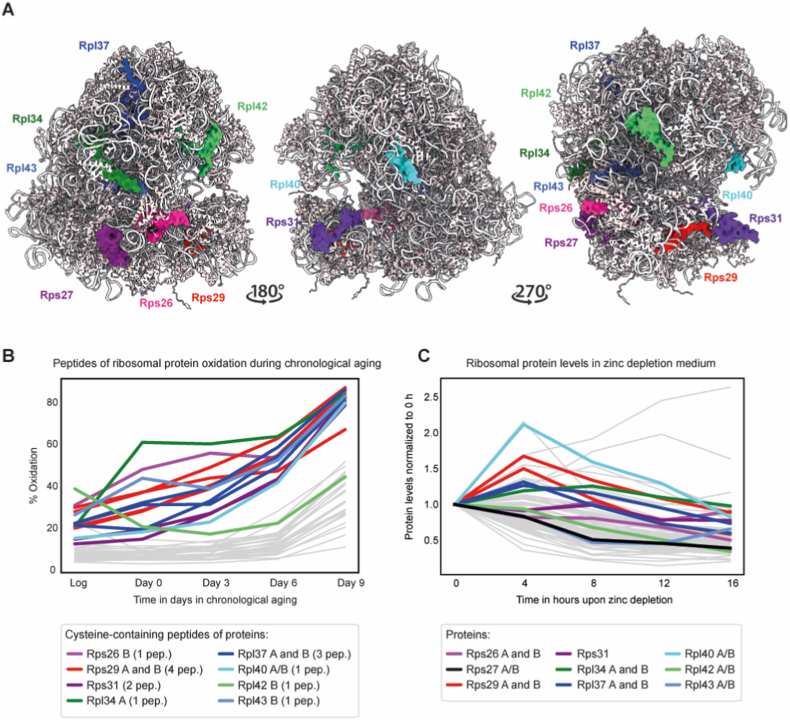
Oxidation dynamics and zinc depletion response of possibly zinc-binding yeast cytoplasmic ribosomal proteins. **(A)** Three-site structural snapshots of the yeast cytoplasmic ribosome (PDB: 4U3M) highlighting ribosomal proteins that contain at least one CxxC motif, indicative of zinc-binding capability. **(B)** Time series (in days) showing changes in reversible oxidation level (%) of cysteine-containing peptides from ribosomal proteins during chronological aging [from Jonak et al. [19]]. Peptides originating from cytoplasmic ribosomal proteins with at least one CxxC motif are colored. Peptides corresponding to paralogous ribosomal proteins (A and/or B) are labeled accordingly; if peptides could not be distinctly assigned to either paralog, they are labeled as “A/B”. *Log* – logarithmic phase; *pep*. – number of quantified peptides. **(C)** Time series (in hours) of changes in ribosomal protein abundance in zinc-depleted growth conditions [from Wang et al. [46]]. Ribosomal proteins containing at least one CxxC motif are colored. Paralogous ribosomal proteins (A and/or B) are labeled accordingly; if peptides could not be distinctly assigned to either paralog, they are labeled as “A/B”.

Among the ribosomal proteins that decrease in abundance in zinc-deficient medium, six exhibit a double CxxC or CxxxC motif: Rps29B (paralog A decreasing with a lower fold change), Rpl37A (paralog B decreasing with a lower fold change), Rpl42B (or A), Rpl43B (or A), and Rps27B (or A). This represents a high fraction of ribosomal proteins with this characteristic, considering the total number of these proteins within the yeast proteome is equal to 9 and 17 if counting paralogs: Rps26A and B, Rps27A and B, Rps29A and B, Rpl34 A and B, Rpl37 A and B, Rpl42A and B, Rpl43 A and B, and ubiquitin-ribosomal proteins Rps31 and Rpl40A and B (Fig. 8A). These proteins form part of the cytosolic ribosome, but do not exhibit a common specific localization within the ribosomal structure. Some are at the surface of the ribosome, like Rps26 or Rps27, while others, like Rpl37, are buried within. The cysteine residues coordinating zinc either take part in the interaction with other ribosomal proteins or rRNA within the ribosome structure (i.e., Rps26, Rps29, Rpl37, Rpl43) or are directed towards the outside of the structure (i.e., Rps27 and Rpl42) (Supplementary Figure 10). Regardless of their location within the ribosome, cysteine sites are predominantly redox-sensitive during the early stages of aging [19].

The CxxC motif-containing cytosolic ribosomal proteins do not follow the typical pattern of oxidation of other ribosomal proteins, having an increase in oxidation level already at day 3 of chronological aging (Fig. 8B; exception: Rpl42B). The same highly oxidized proteins respond similarly to zinc deficiency, however, exhibiting a pattern consistent with other ribosomal proteins that lack a CxxC motif (Fig. 8C). Notably, proteins such as Rps29, Rpl37, and Rpl40 exhibit an immediate increase in abundance within 4 h of shifting to zinc-depleted medium, followed by a steep decrease thereafter. While the reason for this transient increase remains unclear, it may reflect a compensatory transcriptional or translational response intended to sustain ribosome function before the long-term decline caused by sustained zinc limitation and oxidative damage. This aligns with observations that cytosolic ribosomal proteins whose abundance decreases upon zinc depletion undergo increased oxidation during aging.

## 3 Discussion

This study investigates the hypothesis that zinc-binding ribosomal proteins oxidized during early aging serve as evolutionarily conserved zinc buffers or reservoirs, particularly within eukaryotic species. We demonstrate a correlation between zinc-binding proteins and the presence of redox-sensitive CxxC motifs across various lineages. By analyzing proteome oxidation data from budding yeast during chronological aging and oxidative stress, we identified primary oxidation targets, specifically pinpointing nine ribosomal proteins that may respond abruptly to fluctuations in ROS. Phylogenetic and structural evidence further reveal that the CxxC motifs in these nine proteins are evolutionarily locked to maintain zinc coordination. Finally, we explore the mechanical consequences of this sensitivity: molecular dynamics modeling demonstrates that oxidation disrupts the coordination geometry of the zinc-binding pocket. The disruption reduces binding affinity for zinc ions and triggers local protein unfolding. This may lead to further protein degradation, explaining the decrease in protein abundance observed during zinc-limiting conditions and suggesting a possible evolutionary strategy for managing cellular zinc resources under stress.

Zinc supports crucial cellular functions, including proteostasis, DNA repair, and stress resistance across diverse species. Consistent with other reports, the expansion of ZFPs correlates with an increase in genome size and body complexity. This expansion may provide a more complex control over gene regulation, genome stability, and cellular processes essential for the development of multicellular organisms [62,63]. Proteins that bind zinc are essential for maintaining cellular homeostasis and response to environmental changes [30,64].

We propose that ribosomal proteins together with metallothioneins and zinc transporters form an integrated network that buffers zinc bioavailability and adapts to changing cellular conditions. Phylogenetic trees constructed from aligned sequences of CxxC motif-containing ribosomal proteins reveal exceptional evolutionary conservation across eukaryotes, with short branch lengths that reflect ∼70–90% sequence identity for core components, which is above the reported average of 60% between yeast and rat [65]. Low RMSDs (0.26-0.96 Å) in structural alignments prove evolutionary pressures lock the CxxC motifs into superimposable 3D templates optimized for tetrahedral zinc ion coordination. High-resolution structural reconstructions validate conservation of CxxC-flanking helices and loops across species, with deviations limited to peripheral regions. These results support minimal divergence in branch lengths and near-identical motif positions, underscoring selective pressure to retain zinc-binding potential in ribosomal evolution. The sequence-to-structure continuity aligns with published work documenting a universal ribosomal core, where critical domains, such as CxxC, are preserved for enhanced functionality [66,67].

Our computational findings align with established redox mechanisms governing zinc-binding sites, where thiol oxidation acts as a molecular switch to eject Zn^2+^ and destabilize protein structure, as seen in metallothioneins and zinc-finger domains. Molecular dynamics simulations of four early-oxidized ribosomal proteins show zinc distances increasing upon oxidation from <3 Å in the reduced state, matching PDB surveys averaging 2.32 Å for zinc-binding pockets to >5 Å upon sulfenylation (steric/electrostatic repulsion) and disulfide formation (rigidifying cysteine pairs), directly causing zinc ejection within a short simulation time. This mechanism is validated structurally by SAP30L C3H1 finger, where H_2_O_2_-induced Cys29-Cys30/Cys38-Cys74 disulfides release Zn^2+^ and abolish DNA/lipid binding [68]. Experimental validation in Rps26 [69] confirms H_2_O_2_ oxidation releases detectable Zn^2+^, while mutation of one of the two cysteines, 23 or 77, to serine abolishes zinc binding entirely. Although eukaryotic 80S cryo-EM/X-ray structures model only reduced cysteines/Zn sites (the authors did not find any structure with oxygen modeled in the cysteine sulfurs in five compared eukaryotic species), possibly due to purifying under reducing conditions, which precludes oxidized/disordered states, these precedents causally link early cysteine oxidation to ribosomal protein destabilization/turnover under oxidative stress during yeast aging.

Under zinc-limiting conditions, cells appear to degrade ribosomes, releasing zinc for redistribution to essential zinc-dependent enzymes and transcription factors ("ribosome as a zinc bank") [70]. In *S. pombe*, zinc deficiency induces selective degradation of ribosomal proteins and upregulation of proteasome and autophagy pathways [71]. Similarly, in *S. cerevisiae*, Wang et al. [46] observed general downregulation of proteins related to translation and ribosome biogenesis. Concurrently, proteins involved in mitochondrial function, proteasome-mediated degradation, autophagy, and stress response pathways increased in abundance, possibly facilitating the recycling of zinc and amino acids from existing proteins and promoting survival under nutrient deficiency and oxidative stress. This dual response, which involves suppression of translation and activation of proteolytic pathways, reflects a conserved cellular adaptation to zinc deficiency, ensuring that zinc is preferentially allocated to essential enzymes and transcription factors critical for stress resistance and DNA repair. Furthermore, bioavailable zinc ions induce metallothionein synthesis via metal-responsive transcription factor-1 (MTF-1), while NRF2 activation upregulates metallothionein expression, collectively buffering zinc and redox balance [72,73].

In zinc-deficient conditions, eukaryotic ribosomal protein abundance changes occur, but the data on a possible transcriptional regulation remain limited. An RNA-seq study performed in mice shows no significant alterations in ribosomal protein transcript levels under zinc starvation [74]. In bacteria *Bacillus subtilis*, the Zur repressor senses intracellular zinc scarcity and activates operons encoding zinc-lacking ribosomal protein paralogs, such as non-zinc-binding bL31B replacing CxxC motif-containing bL31 [75,76]. This facilitates ribosome remodeling to release bound zinc and prioritize essential translation. Such Zur-mediated transcriptional switching exemplifies an oxidation-independent strategy. In eukaryotes, the Zap1 transcription factor primarily activates zinc uptake and homeostasis genes, like zinc transporter ZRT1, during zinc deficiency [77]. A potential mild repression of selected ribosomal protein genes (RPL27B, RPL34A) lacking canonical zinc-responsive elements (ZREs) has been reported, though the effect size remains modest [78]. Additional data on eukaryotic ribosomal protein transcripts during zinc starvation are needed, as current evidence remains limited.

Oxidation of ribosomal proteins may also trigger specific stress pathways independent of zinc metabolism to respond to changes in environmental conditions. For example, it may trigger selective ribosome repair pathways that involve chaperone-directed recognition and replacement of oxidized protein to maintain ribosome integrity and function during stress conditions [69]. Overoxidation of non-zinc-binding ribosomal proteins, like Rpl10, may trigger ribosome stalling, activating stress surveillance pathways, like the ribotoxic stress response, which engages MAP kinases to modulate translation and promote cell survival [79,80]. Notably, in zinc-binding ribosomal proteins, cysteine oxidation could concomitantly release zinc ions, linking these independent stress pathways to disrupted zinc homeostasis during aging and oxidative challenges.

In conclusion, maintaining zinc homeostasis through coordinated regulation of zinc reservoirs and redox-sensitive pathways may represent an important mechanism influencing aging and proteostasis across species. Reversible oxidative modifications, such as disulfide formation and sulfenylation, could modulate zinc bioavailability and affect the function of zinc-binding proteins and transporters, potentially promoting zinc redistribution and activation of stress responses. Within this framework, highly abundant ribosomal proteins may function as potential redox-sensitive zinc buffers that contribute to cellular zinc trafficking and redox regulation of the proteome during aging across eukaryotes. However, the mechanistic link between ribosomal protein oxidation, zinc redistribution, and aging phenotypes remains to be fully established. Direct functional evidence demonstrating the inter-molecular zinc transfer from ribosomal proteins to, for example, catalytic centers of apo-enzymes in response to age-related stressors has yet to be experimentally validated. Although our observations are consistent with this model, the present study does not directly determine whether these oxidative modifications play a causal role in aging processes or instead reflect broader redox alterations associated with cellular aging. Future functional studies, such as measurements of translational efficiency, intracellular zinc dynamics, and their correlation with oxidation levels in aging models, will be necessary to clarify the biological relevance and potential causal contribution of these mechanisms.

## 4 Methods

### 4.1 Preparation of proteomes’ files

UniProt Knowledgebase (uniport.org) was searched for Reference Proteomes and Unreviewed Proteomes (UniProtKB/TrEMBL) of 64 chosen species, representatives of three domains of life: Archaea, Bacteria, and Eukaryota (see Supplementary Material: “Selected species”). Files containing reviewed and unreviewed entries were downloaded on 24.03.2025 with various annotations chosen, among others, amino acid sequences, Gene Ontology terms, annotations for cofactor binding, keywords, binding sites, etc. All proteomes were then filtered to avoid protein redundancy. Proteins annotated as “fragments” and proteins with duplicated sequences were removed. Only canonical forms of proteins were kept (isoforms removed). Proteins with the same gene name and EC number were removed. If reviewed and unreviewed forms of particular proteins were present, only the reviewed were kept. If no reviewed entry was present, only the longest-sequence entry (longest isoform) was kept. Due to the applied filtering process, the final proteome datasets may be smaller than the corresponding reference proteomes. Importantly, the filtering approach does not impact the overall analysis or the conclusions of this study. Instead, it facilitates an unbiased comparison by preventing the duplication of identical amino acid sequences across proteomes.

### 4.2 Annotations for zinc binding

To identify zinc-binding proteins, we used UniProtKB annotations: “Gene Ontology (molecular function)” (searched for “zinc”), “Cofactor” (searched for “Zn”), “Binding site” (searched for “Zn”), “Zinc finger” (search for presence), “Keywords” (searched for “zinc” and “zinc finger”). Additionally, datasets from Andreini et al. [45] and MetalPDB [32] were used for selected species. Datasets containing information on zinc-binding according to structures from PDB was retrieved from the manuscript (Andreini et al.) and MetalPDB website (on 10.06.2025). Based on information from UniProtKB column containing PDB IDs for each protein in filtered proteomes, information from Andreini et al. and MetalPDB on zinc-binding, the function of the zinc within protein, and amino acids involved in zinc binding were matched. Information on general zinc-binding content from available structures in PDB was obtained from ZincBind database [31] on 22.05.2025. For the *S. cerevisiae* proteome, data on annotated and predicted zinc-binding proteins were retrieved from Wang et al. [46].

### 4.3 Datasets used in this study

Datasets on proteome-wide reversible thiol oxidation in different species were obtained based on Jonak et al. [19]. These data, covering chronological aging in four species, were derived from mass spectrometry approaches using the OxICAT method [41] for *S. cerevisiae* (haploid wild-type strain YPH499 [19]), *C. elegans* from Knoefler et al. [104], and *D. melanogaster* from Menger et al. [105], and the CPT-based method for *M. musculus* from Xiao et al. [16]. The use of Jonak et al. as a primary source for yeast aging was prioritized due to higher peptide coverage compared to other available datasets on redox-proteomics during chronological aging in yeast (DBY749 strain) from Brandes et al. [42]. All identified proteins were mapped onto filtered proteomes of the respective species to identify zinc-binding and CxxC motif-containing proteins.

To evaluate redox responses under stress, datasets on the *S. cerevisiae* proteome during various stress conditions in proliferating cultures were obtained from Topf et al. [18]. The study utilized. YPH499 strain of *S. cerevisiae,* and the reversible thiol oxidation was determined using the OxICAT method. The integration of the datasets on thiol oxidation during aging and oxidative stress conditions in *S. cerevisiae* relied on the use of inherently normalized values, expressed as the percentage of TCEP-reversibly oxidized cysteine-containing peptides within each sample. No additional cross-experimental normalization was performed between independent datasets to avoid artifacts; however, for comparative analysis, data were normalized to their respective internal controls: the wild-type logarithmic phase for stress and mutant conditions [18] and the early logarithmic phase for chronological aging [19]. Positions of redox-sensitive cysteines were retrieved for each quantified peptide and mapped to each other to correlate the redox response.

Zinc-binding capability in these datasets was obtained from annotations retrieved from UniProtKB, Andreini et al. [45], MetalPDB [32], and Wang et al. [46] datasets.

Data on changes in protein abundance upon zinc depletion were retrieved from Wang et al. by mapping protein names to an expanded yeast filtered proteome of 6011 proteins. This experiment specifically compared proliferating cells of *S. cerevisiae* BY-series strain (BY4741, BY4742, BY4743) in the logarithmic phase, grown in low-zinc medium with the addition of 1 μM ZnCl_2_ for the zinc-depletion dataset and addition of 100 μM ZnCl_2_ for the zinc-repletion dataset. Although different yeast strains were utilized in our study, their shared S288C genetic background ensures biological comparability.

### 4.4 Functional enrichment analysis

Enrichment of terms for zinc-binding proteins was performed using DAVID 6.8 [davidbioinformatics.nih.gov, [81,82]]. Annotations selected for analysis included: Functional Annotations UniProt (UP_KW) Biological Process and Cellular Component, Gene Ontology (GOTERMS DIRECT) for Biological Process and Cellular Component, and KEGG PATHWAY. Functional Annotation Clustering was used with classification stringency set to medium and default options. For analysis of zinc-binding proteins across species, we used as background the entire filtered proteomes, while the foreground consisted of proteins annotated as zinc-binding. For analysis and visualization, clusters with enrichment score above 0.5 were considered (exception: *M. jannaschii*). Terms identified in clusters were categorized into Functional Categories using the artificial intelligence language model Perplexity (www.perplexity.ai), for better clarity. Classifications and separate clusters can be accessed in Supplementary Tables 2 and 3 For analysis of zinc-binding proteins in the yeast redoxome during chronological aging, we used as background all proteins with cysteine-containing peptides grouped within clusters A-D (early and middle aging) and within clusters E-H (late aging), while as foreground proteins identified in these clusters as binding zinc. For analysis and visualization, representative terms with the highest gene number and lowest p-value of the top five annotation clusters (Functional Clustering) according to the highest enrichment score.

### 4.5 Motif analysis

Motif analysis was performed using pLogo [plogo.uconn.edu, [83]]. Zinc-binding amino acids according to UniProtKBa annotations “Binding site” were extracted from the sequences of filtered proteomes of chosen model organism species. Peptides centered ±6 amino acids around each zinc-binding amino acid were submitted for analysis with the background set to the available proteome of the respective species, and redundancy was removed from the background and the foreground. Sequences with zinc-binding residue closer to the START or STOP codons than 6 amino acids were removed from the analysis. Amino acids with p-values <0.05 were considered significant.

### 4.6 Analysis of ribosome structures

Structures of the ribosomes were downloaded from Protein Data Bank (PDB) and analyzed using ChimeraX 1.9 [84]. Structures used: 4U3M (yeast), 9BH5 (worm), 6XU8 (fruit fly), 7CPU (mouse) and 9PA7 (human).

### 4.7 Molecular dynamics simulations

For molecular dynamics (MD) simulations, individual yeast ribosomal proteins Rps26B, Rps31, Rpl34A, and Rpl37A were extracted from the 4U3M structure. Ribosomal proteins were extracted along with a zinc atom bound to the cysteine pockets. For Rpl34A, which lacked a zinc coordination center in the original yeast structure, the zinc-binding site was predicted using Metal3D [52] under default parameters. The simulations were performed on isolated ribosomal proteins without the context of the entire ribosome, in which they were structurally solved.

Simulation systems were prepared using the CHARMM-GUI platform and the QM/MM Interfacer [[55], [56], [57]]. To investigate the structural impact of thiol redox states, three distinct chemical environments were modeled for the zinc-coordination centers: (1) reduced (control), where cysteine residues were modeled as deprotonated thiolate anions using the CYM patch, (2) sulfenylated (-SOH), where oxidized cysteines were modeled using the CSO patch, (3) disulfide bonded (-S-S-), where covalent linkages were specified based on predictions from the Scratch Protein Predictor [59]. Additionally, Rps26B was simulated without zinc ion with CSO patch (sulfenylation). Solvation and neutralization were performed using a Monte-Carlo ion placement method with a KCl salt concentration. The CHARMM36 m force field was employed for all macromolecular components.

A hybrid quantum mechanics/molecular mechanics (QM/MM) approach was utilized to accurately describe the zinc coordination sphere. The QM region was defined to include the coordinating cysteine sidechain atoms: Cβ, Hβ1, Hβ2, and Sγ1, additionally with OΔ and HΔ for –SOH. The zinc ion Zn^2+^ (ZN2) was chosen for simulations with zinc. Calculations were performed using the SQUANTUM module within the CHARMM package. The semi-empirical AM1 method was employed with an H-link QM-Link atom treatment to handle the QM/MM boundary. System equilibration was conducted in the NVT ensemble, followed by production dynamics in the NPT ensemble at 303.15 K. To ensure numerical stability within the hybrid interface, SHAKE constraints were applied to the MM region with a standard timestep, while a specialized 1.0 fs timestep was utilized for the QM/MM integration.

Energy minimization and molecular dynamics modeling were performed on a cloud-based simulation platform utilizing the OpenMM engine and CHARMM force field files [58]. To accurately capture the metal-coordination dynamics, the integration script was modified to incorporate specific zinc-binding features (code available at: github.com/katarzynajonak/Zinc-Restraints-for-MD-production), containing zinc restraints. The system underwent initial equilibration starting with 10,000 steps of energy minimization, followed by a simulation phase conducted at 298 K and 1 bar with an integration timestep of 2.0 fs, a duration of 0.1 ns, and a force constant of 5000 kJ/mol/nm^2^. For the production MD phase, simulations utilized an integration timestep of 2.0 fs with a stride time of 0.5 ns per stride. For the simulation of Rps29B without zinc ion, the force constant was set to 500 kJ/mol/nm^2^ for the energy minimization step, and for the production MD phase stride time was set to 2 ns per stride with 5 strides in total (simulation of total 10 ns).

Structural analysis and final coordinate visualization were performed using UCSF ChimeraX v1.9 [84], while trajectory processing and dynamic monitoring were conducted using VMD v1.9.4a53 [85].

### 4.8 Calculation of SASA

Solvent accessible surface area (SASA) was calculated utilizing algorithms of relative SASA calculations [86] in Python version 3.9. Shrake-Rupley algorithm was utilized [87] as implemented in MDTraj [88], using a probe radius of 1.4 Å. Environmental shielding was assessed by calculating the atomic SASA of the full protein and summing the contributions of the CSO residues (shielded SASA). To determine the intrinsic expansion of the cluster, the CSO residues were isolated, and SASA was recalculated in the absence of the protein matrix (maximum/”isolated” SASA). Relative exposure was defined as the ratio of shielded to maximum SASA. Code is available at github.com/katarzynajonak/SASA-calculation-for-cysteine-sites. Results were compared to ChimeraX SASA calculation algorithm.

### 4.9 Sequence alignment and phylogenetic analysis of ribosomal proteins

Clustal Omega [68] was used for sequence alignment and preparation of phylogenetic trees for CxxC-motif containing ribosomal proteins of selected species. Default options were used. Newick files were submitted to iTOL [89], and the branch lengths were used to scale the tree.

### 4.10 Phylogenetic tree preparation

Newick files for 64 species were generated using timetree.org [90], and the time tree view was adapted using iTOL itol.embl.de [89]. *Nitrosopumils martimus*, *Ramazzottius varieornatus* (tardigrade), and *Schmidtea mediterranea* (planarian) were not depicted due to the inability of timetree.org to include the species in the analysis. Newick file branch lengths represent relative divergence times between species or nodes. In iTOL, these branch lengths are used to scale the tree for visual interpretation of the relative distances between species. The "Tree scale: 1000″ in iTOL indicates how these branch lengths map visually in the tree display (e.g., that a branch length of 1000 units corresponds to the length of the scale bar; tree scale = 1000 corresponds to 1000 million years).

### 4.11 Bioinformatic analysis and visualization

Analyses presented in the study were performed using Python version 3.9. Figures were prepared using Python libraries matplotlib 3.9.0 and seaborn v0.13 and Adobe Illustrator.

### 4.12 AI tools

Artificial intelligence tools Perplexity (www.perplexity.ai) and Grammarly (grammarly.com) were used to ensure the accuracy and correctness of the grammar in some sections of the manuscript. Perplexity was used to categorize functional enrichment terms (see “Functional enrichment analysis”).

### 4.13 Limitations of the analysis

#### 4.13.1 Proteome files

The limitation of meta-analyses of multiple species relates to the number of manually curated and annotated proteins that contain experimentally verified or carefully evaluated functional information. Only a handful of proteomes are marked as “reviewed”, making it difficult for researchers to perform high-quality bioinformatics analyses.

#### 4.13.2 Zinc-binding annotations

Analyzing the zinc-binding proteome using existing datasets from UniProtKB, GO annotations for molecular functions, and data on experimentally solved structures of proteins from the PDB is limited. Completeness and accuracy of zinc-binding annotations vary widely across datasets: UniProtKB entries and GO terms may lack consistent or experimentally validated zinc-binding information, often relying on indirect evidence. Structural data in the PDB, used, among others, in ZincBind or MetalPDB, are biased towards proteins that have been successfully crystallized, which represents only a small subset of the total proteome and species. Zinc-binding sites may be missed due to conditions that do not preserve metal coordination. Additionally, the dynamic nature of metal binding and the presence of transient or weakly bound zinc ions complicate detection in static structural snapshots.

## Funding

This work was supported by the 10.13039/501100004281National Science Centre Poland, SONATA grant no. 2022/47/D/NZ3/01740 to KJ and 10.13039/100025296OPUS grant no. 2022/45/B/NZ1/03714 to UT. KJ was additionally supported by the 10.13039/501100003043EMBO Postdoctoral Fellowship grant no. ALTF 82-2022.

## CRediT authorship contribution statement

**Katarzyna Jonak:** Conceptualization, Data curation, Formal analysis, Funding acquisition, Investigation, Methodology, Software, Validation, Visualization, Writing – original draft. **Ulrike Topf:** Conceptualization, Funding acquisition, Project administration, Supervision, Validation, Writing – review & editing.

## Declaration of competing interest

The authors declare that they have no known competing financial interests or personal relationships that could have appeared to influence the work reported in this paper.

## Data Availability

The data used for this research are available in the manuscript and the supplementary material.
