# Supplementary material for: Integrated analysis of the redoxome and zinc proteome links ribosomal protein oxidation to zinc homeostasis
Source: Redox Biol. 2026 Apr 2;93:104152. doi: 10.1016/j.redox.2026.104152 (PMC13090322; doi:10.1016/j.redox.2026.104152)
Supplement: Multimedia component 6 [file mmc6.pdf]

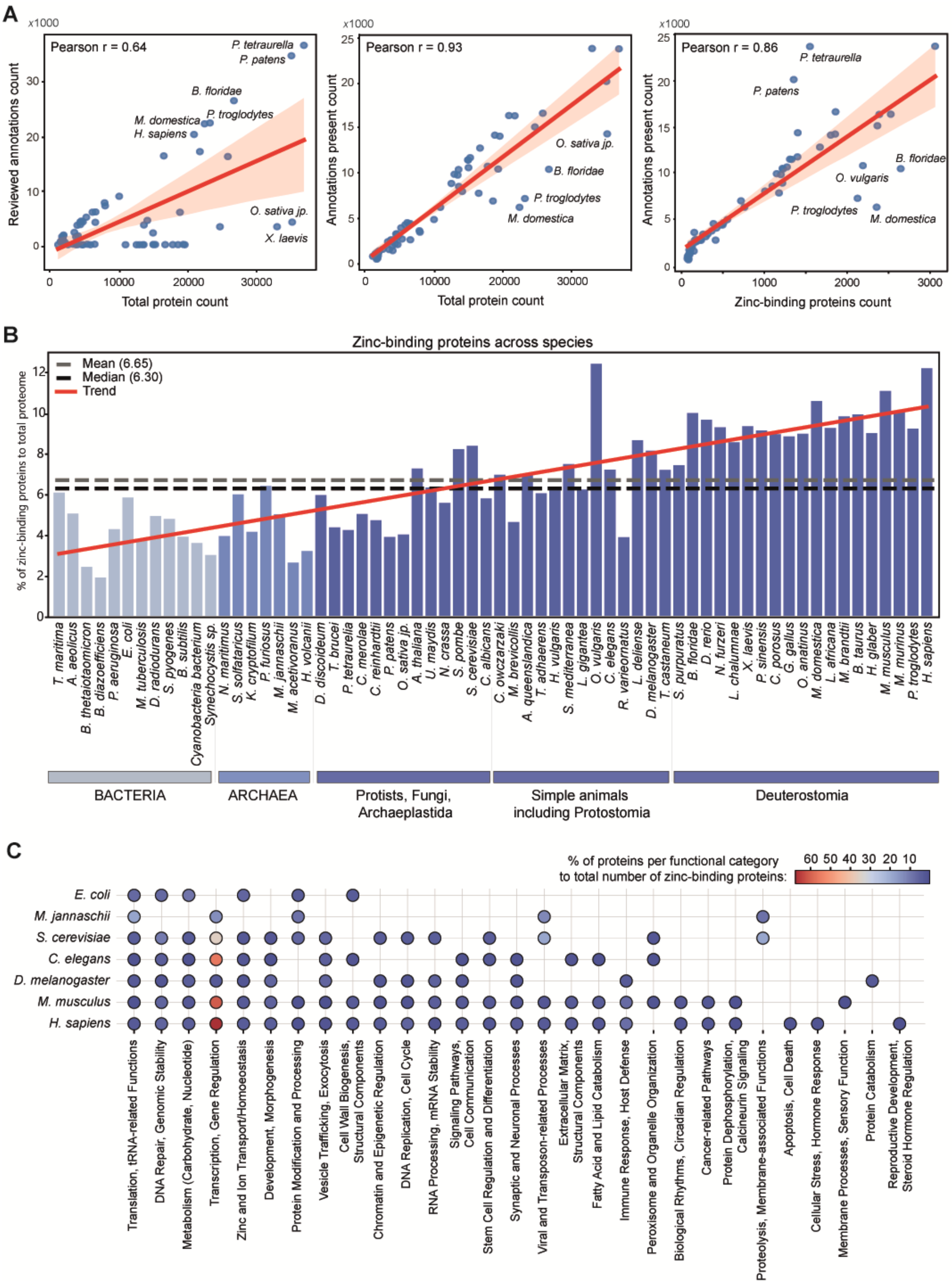


**Sup. Figure 1 (related to Figure 1): Quality assessment and comparative analysis of zinc-binding proteins across the tree of life.**

**(A)** Quality assessment of zinc-binding protein annotations from UniProtKB across studied species. The left and middle panels depict correlation plots between the number of proteins in each species' proteome and the number of proteins with reviewed annotations (left) or any annotation present across species (middle). "Annotation present" refers to any entry in UniProtKB fields including "Gene Ontology (molecular function)", "Cofactor", "Binding site", or "Zinc finger". The right panel shows the correlation plot between the number of zinc-binding proteins and the number

of proteins with annotation present. Blue dots – number of a single species; red line – the trend line fitted by linear regression; *r* – Pearson's correlation coefficient. **(B)** Fraction of zinc-binding proteins across 64 species. Red line – the trend line fitted by linear regression; black dashed line – median across all species; grey dashed line – average across all species. **(C)** Functional enrichment analysis of zinc-binding proteins in representative species of the three domains of life. Each dot corresponds to a functional category cluster enriched among zinc-binding proteins. Dot colors indicate the normalized number of annotated genes/proteins per category relative to the total zinc-binding proteins in that species. Absence of a dot indicates no significant enrichment for that category.

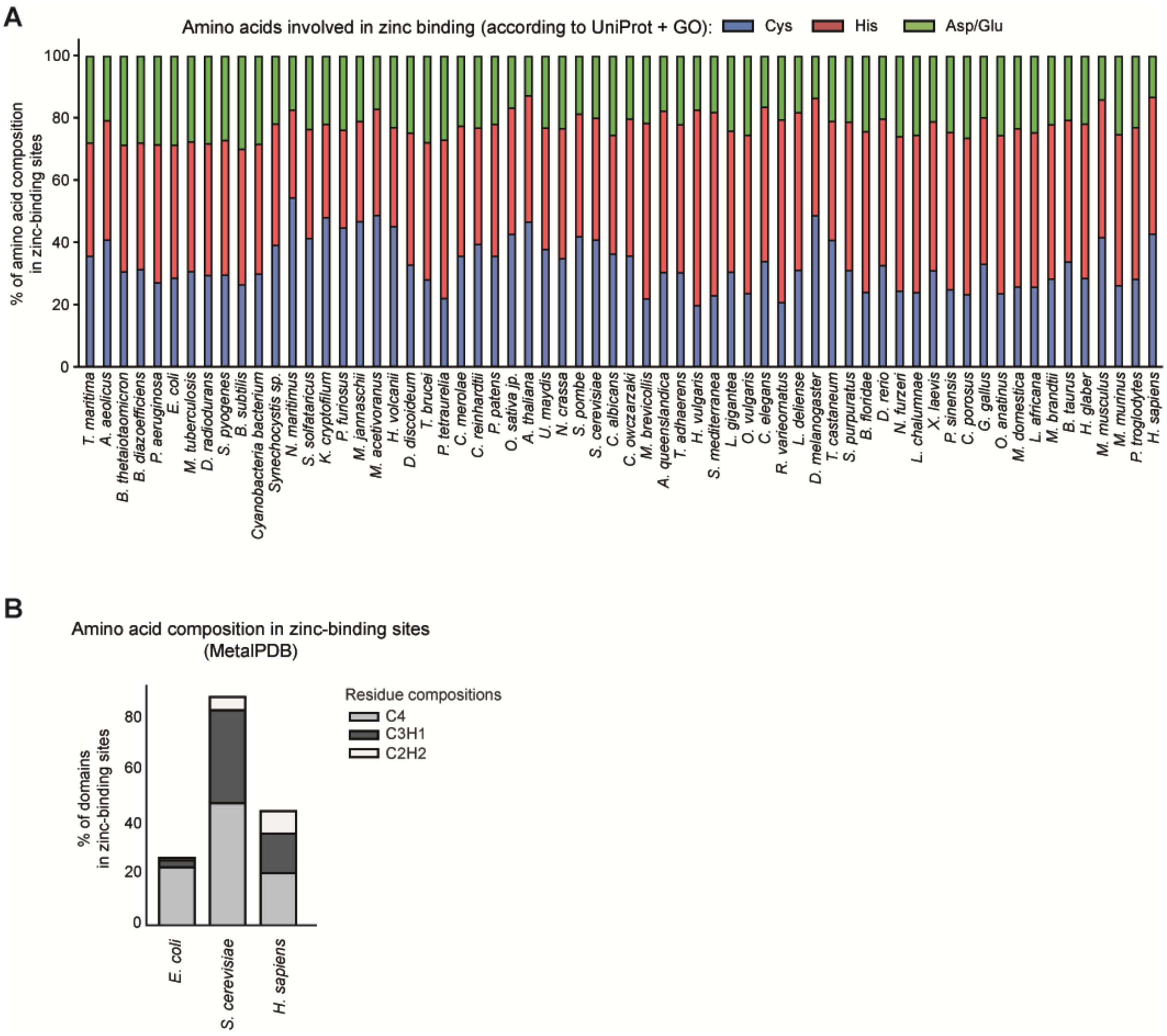


**Sup. Figure 2: Amino acid composition of zinc-binding sites across species.**
**(A)** Proportion of zinc-binding proteins containing at least one specified amino acid involved in zinc coordination, normalized to the total number of zinc-binding proteins annotated in UniProtKB "Binding site" dataset. The most common amino acids involved in zinc binding are depicted: cysteine (Cys, C), histidine (His, H), aspartic acid (Asp, D), and glutamic acid (Glu, E). The combined fractions of C, H, D, and E were calculated to sum to 100%, and each fraction is plotted accordingly. **(B)** Proportion of proteins harboring cysteine- and histidine-containing zinc-binding domains, normalized to the number of proteins with known domains according to MetalPDB. The most prevalent zinc coordination motifs are depicted: C4 (four cysteines), C3H1 (three cysteines and one histidine), and C2H2 (two cysteines and two histidines).

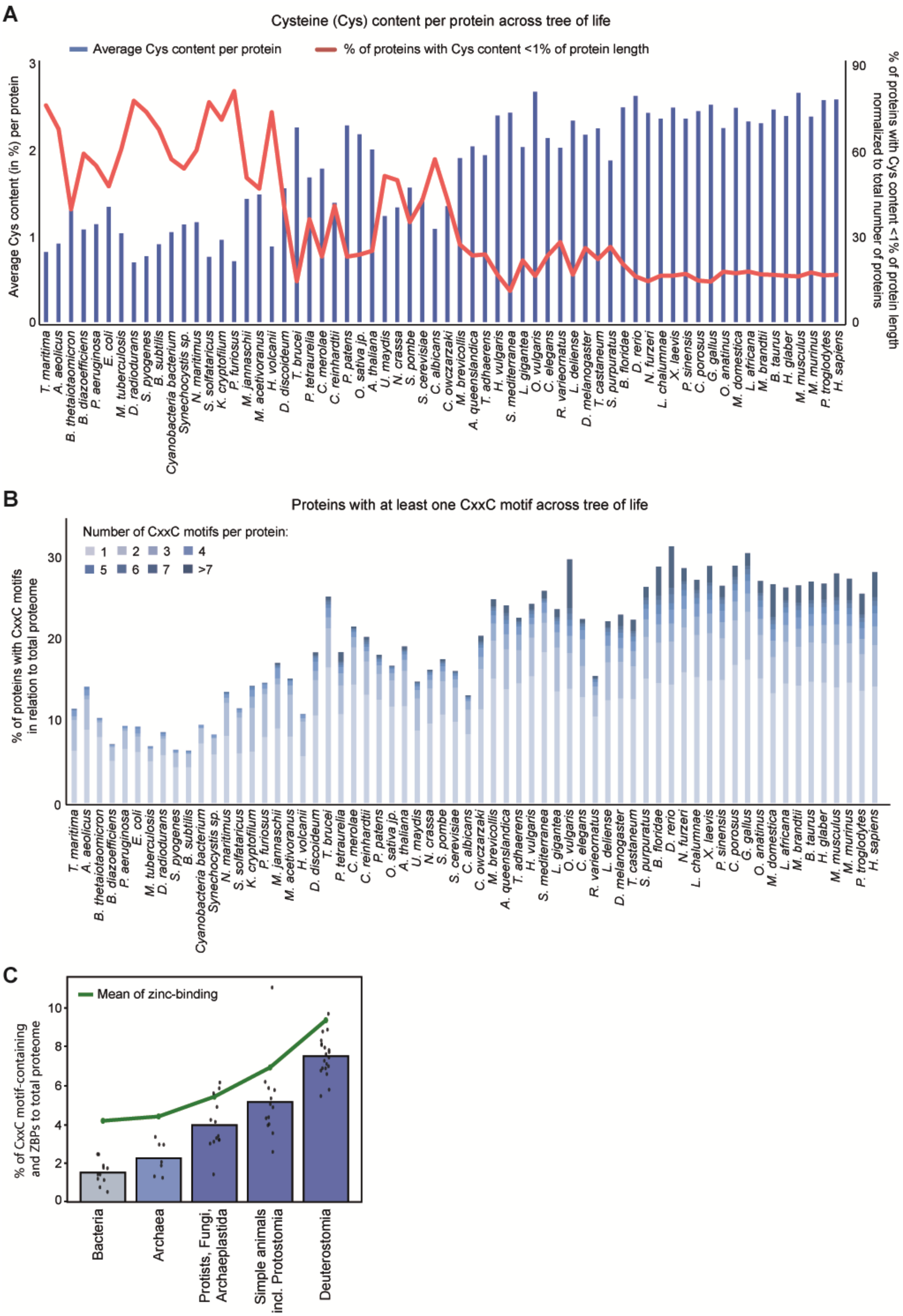


**Sup. Figure 3 (related to Figure 2): CxxC as a zinc-binding motif across species. →**

**Sup. Figure 3 (related to Figure 2): CxxC as a zinc-binding motif across species.**
**(A)** Fraction of proteins containing a particular number of cysteine residues (Cys) normalized to protein length, giving cysteine content in 64 species. Blue bars indicate the average cysteine content per protein (left *y*-axis). Red line indicates fraction of proteins with cysteine content below 1% of protein length, normalized to total number of proteins per species (right *y*-axis). **(B)** Fraction of proteins containing at least one CxxC motif normalized to total protein count in 4 species. Different shades of blue indicate fractions of proteins with a particular number of CxxC motifs found in the analysed amino acid sequences. **(C)** Average proportion of CxxC motif-containing and zinc-binding proteins across proteomes of 64 species. The Eukaryota domain was divided into three groups: Protists, Archaeplastida and Fungi (includes plants and simple eukaryotes), simple animals including Protostomia, and Deuterostomia (includes vertebrates). Black dots – individual species values; red dashed lines – group medians; green line – average fraction of zinc-binding proteins normalized to total proteome count, based on UniProtKB.

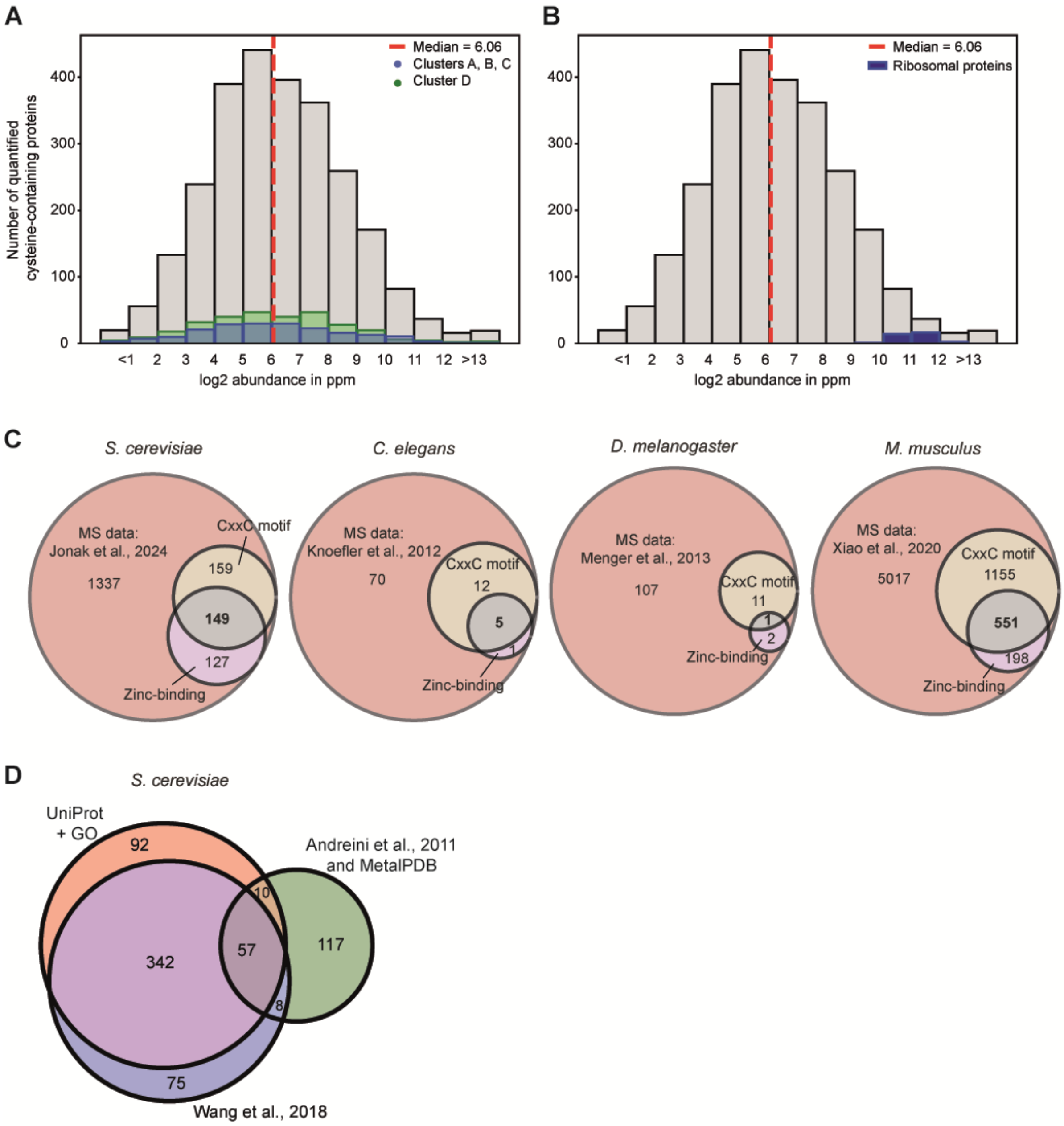


**Sup. Figure 4 (related to Figure 3): Quantitative analysis of cysteine oxidation in proteomes of aging species.**

**(A-B)** Distribution of cysteine-containing proteins identified in the yeast OxiAge study from Jonak *et al.* according to absolute protein abundance. Protein abundance shown in parts per million (ppm) is obtained from pax-db.org for *S. cerevisiae* – whole organism (integrated database with coverage of 96%). Red dashed line is the median value. Additionally, in (A) distribution of proteins assigned to oxidation clusters A-C (blue) and D (green) is shown. In (B), distribution of all quantified ribosomal proteins (dashed blue) is shown. **(C)** Number of CxxC motif-containing proteins and total zinc-binding proteins with quantified reversible oxidation at cysteine residues during aging as determined by mass spectrometry (MS) studies in four species: *S. cerevisiae* from Jonak *et al.*, *C. elegans* from Knoefler *et al.*, *D. melanogaster* from Menger *et al.*, and *M. musculus* from Xiao *et al.* These data are mapped onto filtered proteomes containing proteins with at least one cysteine residue. For yeast, the proteome was expanded to include 6011 proteins previously removed due to redundancy in order to maintain consistency with oxidation data from Jonak *et al.*, which form the basis for subsequent analyses. The proteomes for the other three species are based on filtered datasets without extension. Zinc-binding annotations are taken from UniProtKB. For yeast, zinc-binding proteins include those annotated in UniProtKB, Andreini *et al.*, MetalPDB, and Wang *et al.*, reflecting both broad and zinc-focused annotations for further analysis. **(D)** Number of proteins annotated as zinc-binding in four datasets for yeast *S. cerevisiae*: UniProtKB annotations (“UniProt + GO”), structural annotations from Andreini *et al.* and MetalPDB, and annotations from the zinc-focused publication Wang *et al.* The four datasets provide complementary views of zinc-binding proteins from sequence-based, structural, and literature-curated sources.

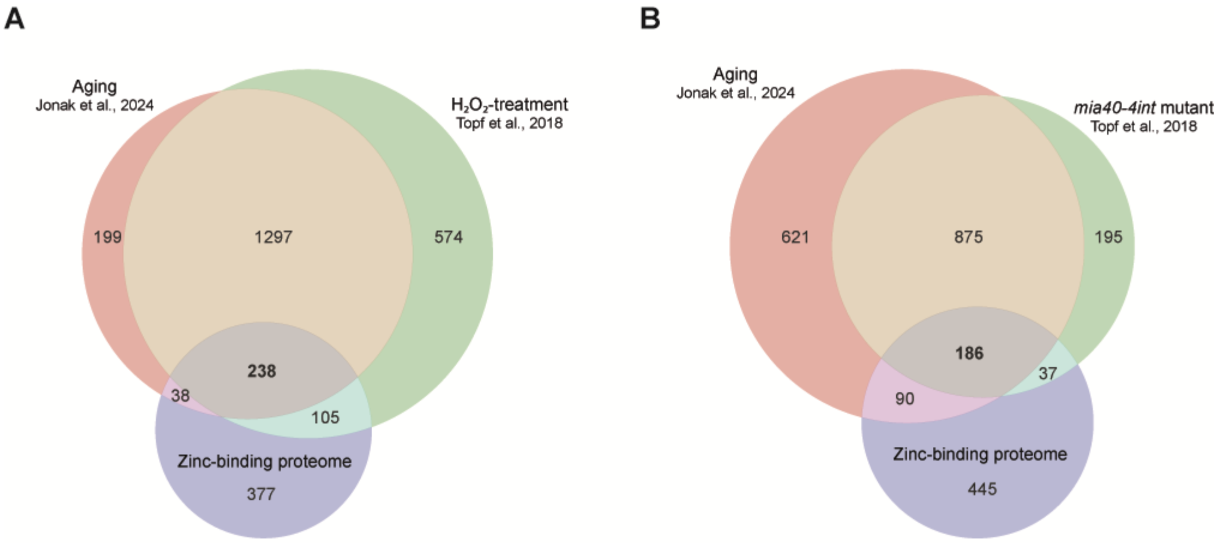


| | Aging<br>Jonak et al., 2024 | $H_2O_2$-treatment<br>Topf et al., 2018 |
|---|---|---|
| Total proteins | 1772 | 2214 |
| Total cysteines | 3564 | 4785 |
| Common proteins | **1535** | |
| Common cysteines | **2663** | |

| | Aging<br>Jonak et al., 2024 | *mia40-4int* mutant<br>Topf et al., 2018 |
|---|---|---|
| Total proteins | 1772 | 1293 |
| Total cysteines | 3564 | 2360 |
| Common proteins | **1061** | |
| Common cysteines | **1499** | |

**Sup. Figure 5 (related to Figures 4 and 5): Zinc-binding proteins sensitive to oxidation during different conditions of oxidative stress.**

**(A-B)** Comparison of the number of zinc-binding proteins quantified as reversibly oxidized during yeast chronological aging (dataset from Jonak *et al.*) and upon $H_2O_2$ treatment (A) or in the mitochondrial import receptor mutant strain, *mia40-4int* (B; dataset from Topf *et al.*) Zinc-binding proteins identified in the $H_2O_2$-treated samples (A) or in the *mia40-4int* mutant condition (B) were mapped to those quantified in the aging dataset to assess overlap and differential redox responses under physiological and acute oxidative stress conditions or under genetic perturbation, respectively. Tables below the diagrams indicate the number of common proteins and cysteine residues among the oxidation datasets.

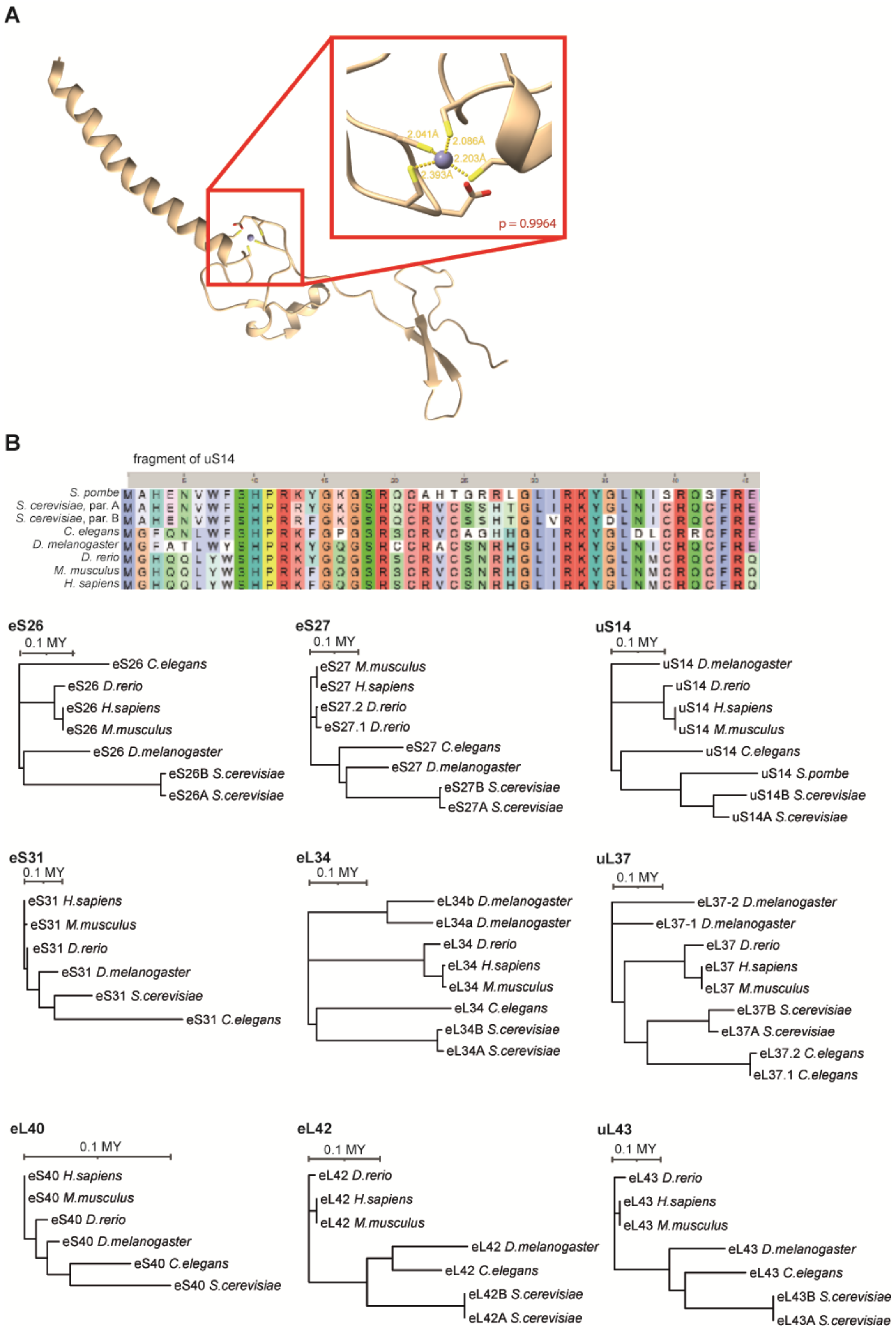


**Sup. Figure 6 (related to Figure 6): CxxC motif-containing ribosomal proteins across species.**
**(A)** Structure of modeled zinc binding in Rpl34A yeast ribosomal protein by Metal3D. Zinc is bound by four cysteine residues with a probability (p) of 0.9964. **(B)** Evolutionary comparison of amino acid sequence of CxxC motif-containing ribosomal proteins. Upper panel shows a representative alignment of the cysteine-containing fragment of uS14 (Rps29A/B in yeast). Lower panel shows phylogenetic analysis of CxxC motif-containing ribosomal proteins. Time scale is given in millions of years (MY).

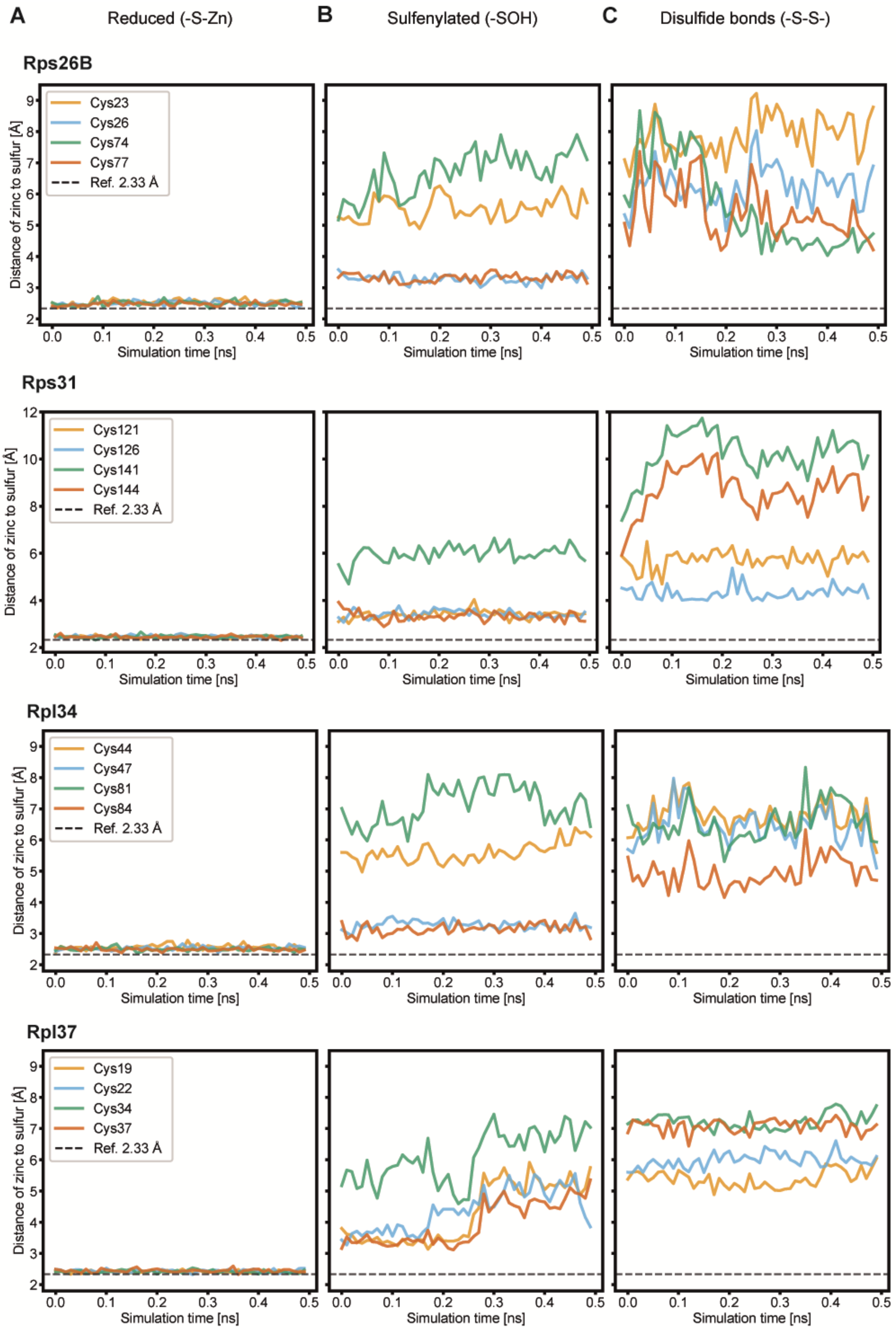


**Sup. Figure 7 (related to Figure 7): Changes in the distance of zinc from sulfur atoms of cysteine residues in short MD simulations.**

Distance in Angstrom (Å) between zinc ion and sulfur atoms of cysteines involved in zinc binding in the reduced thiolate state. Simulation time in nanoseconds [ns] is shown on *x*-axis. Each curve represents distances calculated

for each cysteine. **(A)** Models of zinc binding in structures with deprotonated cysteines (-S-Zn). **(B)** Models of zinc repulsion in structures with sulfenylated cysteines (-SOH). **(C)** Models of zinc repulsion in structures with disulfide bonds (-S-S-). Dashed black lines represents the reference distance of 2.33 Å (average reported zinc-binding distance).

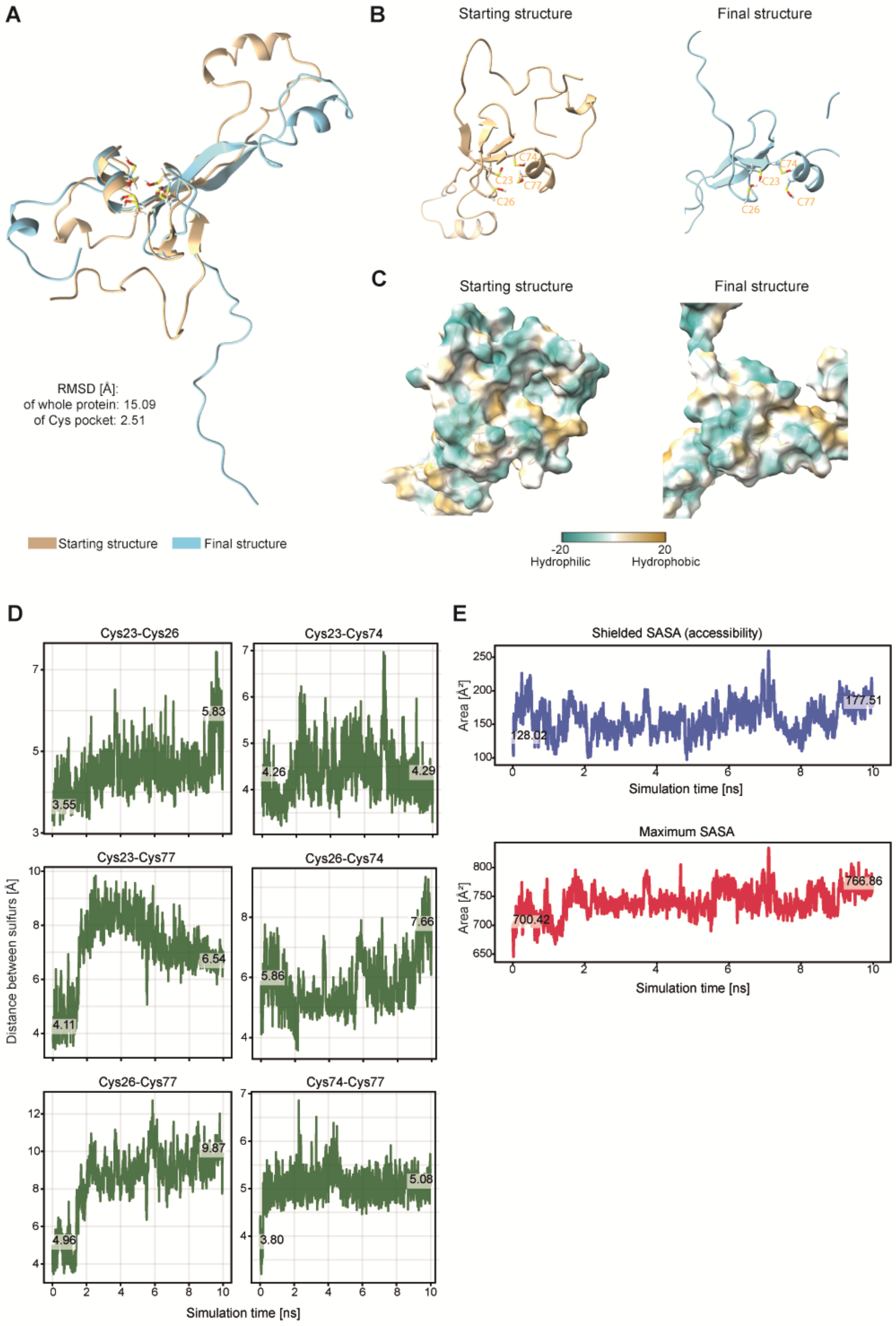


**Sup. Figure 8: Structural changes of ribosomal protein representative, yeast Rps26B, in the absence of zinc upon -SOH modification of cysteine residues. →**

**Sup. Figure 8: Structural changes of ribosomal protein representative, yeast Rps26B, in the absence of zinc upon -SOH modification of cysteine residues.**

**(A)** Superimposed models from MD simulations at the start of the simulation (minimized energy model, beige) and at the end of the simulation (10 ns of MD, blue). RMSD between structures is calculated in Angstrom (Å) for carbon alpha atoms of the whole protein and of the cysteine pocket (+/-6 amino acids surrounding the cysteines). **(B)** Zoom in on the cysteine sites of the simulated protein. **(C)** View on protein surface of cysteine pockets from (B) with hydrophobic patches added from ChimeraX. **(D)** Distance in Å between sulfur atoms of each cysteine pair during simulations. Values at the start and the end of the simulations are written on the plots. **(E)** Solvent accessible surface area (SASA) of cysteine sites during simulations (in $Å^2$ area units). Shielded SASA calculates cysteine residues within the protein environment, while maximum (“isolated”) SASA calculates cysteine residues in an isolated out-of-the-protein state. Values at the start and the end of the simulations are written on the plots.

**A**

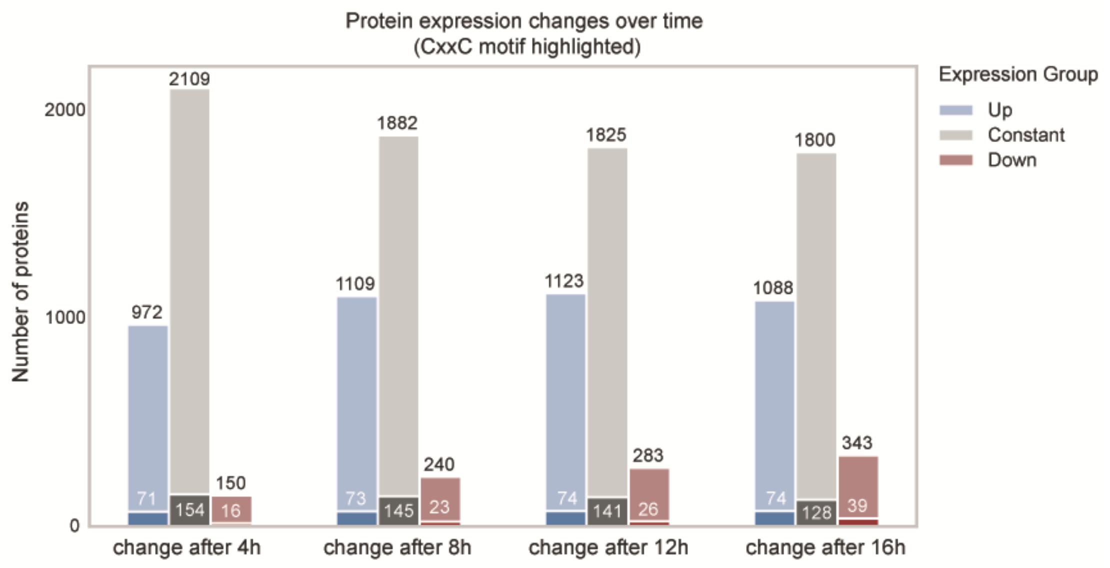


**B**

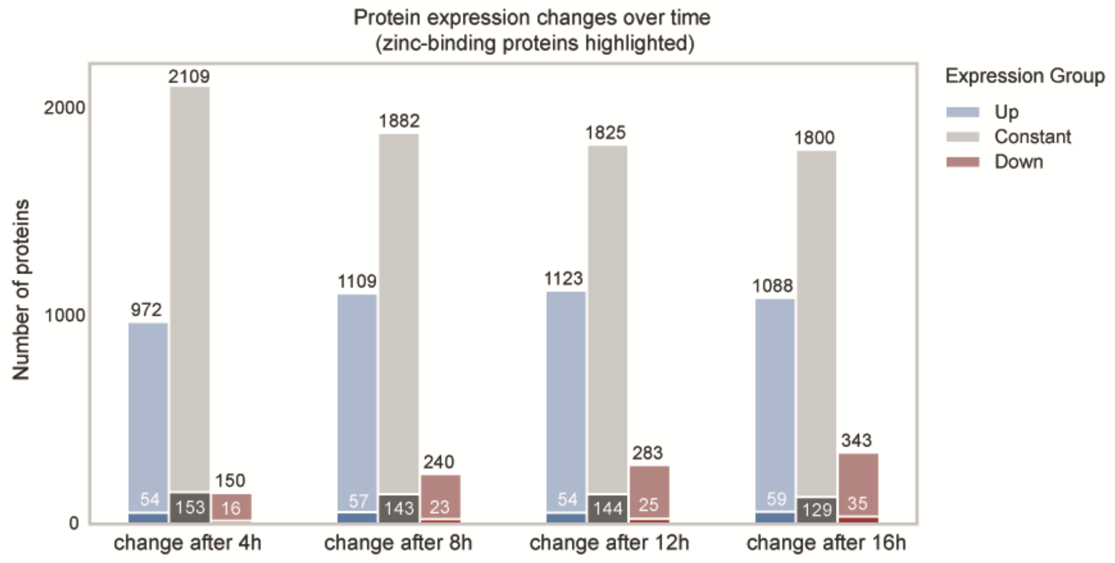


**Sup. Figure 9 (related to Figure 8): Proteomic response to zinc deficiency and the relationship to protein oxidation during yeast aging.**

Data on oxidation during aging is obtained from Jonak *et al.*, while data on protein level changes in zinc-deficient medium is obtained from Wang *et al.* **(A-B)** Distribution of proteins based on their abundance changes at 4, 8, 12, and 16 hours after inoculation into zinc-deficient medium. Changes are shown relative to time point zero. Bar plots indicate proteins whose levels increase ("Up", fold change > 0.5), decrease ("Down", fold change < -0.5), or remain unchanged ("Constant", fold change between -0.5 and 0.5). **(A)** Proteins containing the CxxC motif are highlighted with darker shades. **(B)** Proteins annotated as zinc-binding are highlighted with darker shades.

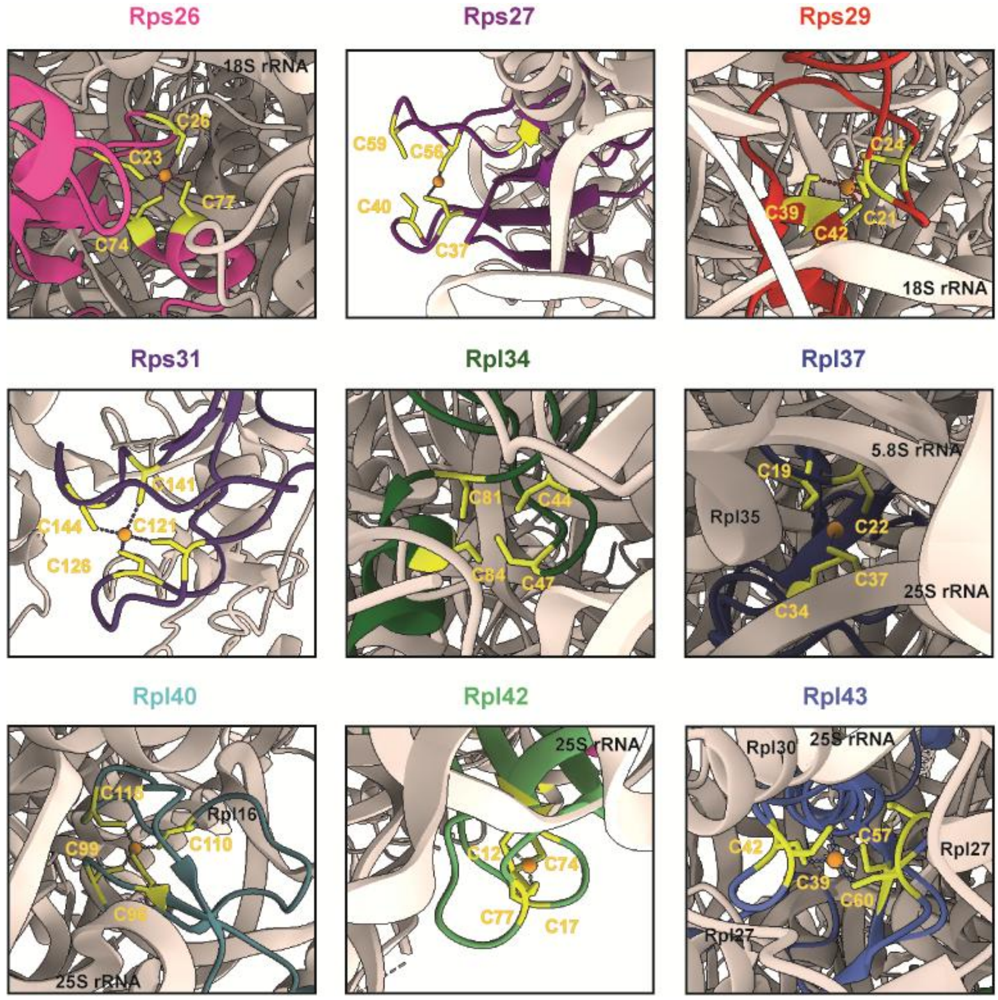


**Sup. Figure 10 (related to Figure 8): Structures of yeast cytoplasmic ribosomal proteins containing the CxxC motif.**
Structural snapshots from the yeast cytoplasmic ribosomal proteins (PDB: 4U3M), highlighting ribosomal proteins that contain at least one CxxC motif, indicative of a zinc-binding feature. Cysteine residues ("C") are shown in yellow and their positions within the protein are labeled. The zinc ion coordinated by these cysteines is shown in orange. Snapshots may include names of nearby ribosomal proteins and/or ribosomal RNA to the depicted CxxC motifs.
